# Clinical Researches on Auscultation of the Respiratory Organs, and on the First Stage of Pulmonary Phthisis

**Published:** 1840-04

**Authors:** 


### BRITISH AND FOREIGN MEDICAL REVIEW,

FOR APRIL, 1840.

### PART FIRST.

### Analytical and Critical Reviews.

### Art. I.

*Recherches Cliniques sur l'Auscultation des Organes Respiratoires, et sur la Première Période de la Phthisie Pulmonaire.* Par Jules Fournet.—*Paris,* 1839. One Vol. In Two Parts. 8vo, pp. 1029.
*Clinical Researches on Auscultation of the Respiratory Organs, and on the First Stage of Pulmonary Phthisis.* By Jules Fournet.

Remarkable as the history of Laennec's immortal discovery is in many points of view, the length of time which elapsed between the first promulgation and establishment of any considerable improvement in his system, forms, perhaps, its most striking peculiarity. Such were the multiplicity, the variety, and the delicacy of the phenomena he described, that, unless in the case of persons singularly gifted by nature, the very task of familiarizing themselves with the physical characters and diagnostic bearing of these was sufficient to absorb, for a length of time, all the keenness of the senses, and exercise all the vigour of the intellect. It would doubtless, too, have seemed a sort of heresy, once the incredulity of ignorance and inaptitude had been overcome, to question the accuracy of what the great discoverer had pronounced to be true, to look beyond the secrets he had chosen to reveal. But the cause which most powerfully contributed to the stability of Laennec's doctrines was of a kind still more honorable to him; for it was none other than the exceeding accuracy of his particular observations: by this was ensured a degree of perfection in his general results, which has rarely attended the first labours of a philosopher in a new path of science. Frequently the power of execution seems, as it were, blunted by splendour of conception; the mind that conceives shrinks from the minute details of practical application. But it was far otherwise with Laennec: each petty detail was followed out with a precision and felicity of observation usually belonging only to the humbler order of minds well fitted to apply the discoveries of others, but themselves deficient in the genius to create.

In proportion, however, as men became familiarized with the details of the new science, and especially after the death of its founder, the spell was dissolved; and the senses sharpened under his tutoring were directed to the detection of flaws in his system. That the result has been of a most valuable kind, that improvements have been made, that some crude observations and hasty generalizations of Laennec have been exposed, and new facts established, is matter of notoriety. The observers, both at home and abroad, to whom we are indebted for these changes, have been contented to follow in the track of Laennec, and without swerving from the method in which he observed the audible phenomena of respiration, to announce such scattered novelties as presented themselves in the course of clinical study. But the case is to a certain degree different with M. Fournet, to whose volume we now propose to draw the reader's attention. Had this observer not perceived the possibility, as he informs us, of pursuing the study of auscultation differently from his predecessors, and of examining its phenomena on new principles, he would not have conceived the hope of enriching the science with important novelties, nor would the present volume have seen the light. What these "principles" are we shall at once inform the reader. M. Fournet, then, has invariably distinguished, both in health and disease, the two murmurs, the inspiratory and the expiratory, accompanying each act of respiration; whereas Laennec habitually spoke of these murmurs as of a single sound. He has, also, minutely analyzed the properties of each of these sounds, and so ascertained the existence and diagnostic importance of several abnormal conditions, which had either escaped, or been imperfectly put to profit by others; and by methodically enquiring, in the instance of every thoracic affection, with which murmur the morbid phenomena of respiration coexist, he has succeeded in recognizing many new signs, and obtained a clearer insight than previous observers into the relations connecting a variety of others, their successive conversion into each other, their natural course, and its accidental interruptions. The general practical result of all this is, it is alleged, a new degree of certainty and precision in the diagnosis, and, consequently, in the prognosis and treatment of pulmonary complaints. But the particular result, that on which M. Fournet's energies have been chiefly expended, is the power of detecting tuberculous disease at an earlier period of its existence than had previously been deemed practicable.

In this statement we have pretty closely followed the author's own enunciation of his achievements. Though his estimate of these may not be distinguished for modesty, the reader will allow that, unless it be most powerfully exaggerated, there must here be materials worthy of the gravest examination—materials of a kind to silence the sophistry of those interested persons, in whose hands, either from prejudice, laziness, or incapability, the stethoscope is destined for ever to remain an *inutile lignum.**

* Auscultation has many avowed adversaries to contend with; these it fears not. The encomiums of its putative friends are, in some cases at least, much more likely to shake its credit. The worst of objections of the class referred to, is that they prove, not the inefficacy of auscultation, but the incapacity of Dr. A. or Mr. B. There are persons who would not be conscious of any more disagreeable auditive impression from hearing two barrel organs play different airs together at the same door, than from hearing "A te O Cara," from the boards of Her Majesty's Theatre, with Rubini for the tenor.

M. Fournet's work is divided into two parts: the first devoted to auscultation generally; the second to the history of the earliest stage of phthisis, its diagnosis, etiology, curability, and treatment. We shall pursue this order in our examination of the volume.

The fundamental distinction between these researches and others on the same subject, consists in the accuracy with which the condition of the expiratory murmur is examined in its normal and perverted states; it is, therefore, not without interest in this place, to trace the progress of knowledge concerning this sound, as well as our author's connexion therewith. The reader need hardly be told, that Laennec in his prolegomena, described in set terms the existence of an inspiratory and expiratory murmur, as accompanying the exit and entrance of air from and into the lungs. Yet, by a strange oversight, the most serious of which he was ever guilty, he speaks, throughout his work, of the respiratory murmur as of a single sound. The authority of his name, as well as some circumstances connected with the phenomenon itself, contributed to keep observers in ignorance of the existence of an expiratory murmur; until the well-known discovery made by Dr. Jackson, in 1832, respecting its distinct occurrence opposite tuberculated lung, directed the attention of numbers to the subject. M. Louis, in particular, recognizing the importance of the new sign, invariably enquired into its condition in all cases of suspected tuberculization; and was gradually led, from extending his enquiries, to the conclusion, that expiration always produces an appreciable, but exceedingly slight murmur in the state of health. The latter fact was by some considered, though, as we shall see, erroneously, to invalidate the importance of Jackson's observation in respect of phthisis. Other French observers came to similar conclusions; and in this country, the existence of an expiratory murmur was formally recognized, and formed the theme of an *ex professo* paper by Dr. Cowan.* Except in the instance of phthisis, the sound does not appear to have been habitually examined in its diseased aberrations; but we may be permitted to remind our readers, that we nearly two years since dwelt on the important information to be obtained from their study in pulmonary emphysema.†

With the knowledge he possessed of these facts, as far as they refer to Parisian practice, it is not a little curious to find M. Fournet recording the "astonishment" he felt, when he commenced his enquiries in 1835, at discovering that two murmurs instead of one accompanied the act of respiration. On Monday, this gentlemen, as yet a student, hears M. Louis and others speak of "prolonged, hard, or bronchial" expiration

No doubt if these persons favoured the world with their lucubrations on music, they would set out, by the aphorism, that the distinction of harmony and discord is mere fudge. But whom would they persuade, unless individuals sharing in their unfortunate style of organization? Neither will they, who are incapable of appreciating the delicate physical signs of disease, persuade those gifted with acuter senses to sacrifice their superiority. It must not be imagined from this that we regard auscultation as only to be compassed by a select few; on the contrary, we believe that there is nothing wanting but the early and proper study of its phenomena, to enable every one, possessed of the average degree of perfectness of the auditory sense, to attain a knowledge of it sufficient for all ordinary practical purposes. Here, as in everything else, there will and must be degrees of excellence.

* Med. Gazette, vol. xviii., p. 332. † Br. and For. Med. Rev., Vol. VI., p. 37.

in the common course of things, and marvels not; on Tuesday, he assumes the character of an original observer, and lo! he is forthwith astounded at hearing two respiratory sounds, because *Laennec* constantly refers to but one. This weak attempt to arrogate to himself what his countrymen would call the *idée mère* of his researches, gives painful evidence of M. Fournet's vanity.

In a somewhat prolix introduction, we are made acquainted with the properties of the respiratory sounds, which it has been the business of this observer to investigate, together with the method followed and precautions used by him in the practice of auscultation. The points which he conceives should be examined in each sound are: 1, *its proper or distinctive character;* 2, *its hardness or softness;* 3, *its dryness or humidity;* 4, *its quality* (timbre*); 5, *its note or tone;* 6, *its intensity;* 7, *its duration;* 8, *its rhythm.* To follow M. Fournet in his remarks on each of these properties is unnecessary, as their nature and importance as signs will appear in our examination of the subsequent sections of the work; but we shall here briefly notice some experiments made with sponge, for the purpose of ascertaining the mode of production of the dry, humid, and bubbling characters.

If a perfectly dried sponge be alternately compressed, and allowed to dilate close to the ear, a sound, varying in delicacy with the fineness of that substance, and distinctly conveying an impression of dryness, is perceived. If the sponge be then wetted, at first slightly, and then to a gradually increasing degree, a sensation of humidity is produced: "the humid character is at first homogeneous, then divides into a number of unequal consecutive sounds; the latter separate from each other, become isolated, and finally produce the sensation of bubbles originating, being developed and bursting." The transition from the slightest to the greatest possible degree of humidity is marked by distinct phases in the audible phenomena produced. But the result may be varied by changing the liquid with which the sponge is imbibed; when water is the fluid employed, the sensation of humidity is simple, uncombined with a character of viscidity, and resembles the respiration of an œdematous lung. If water, slightly charged with blood or gummy matter, be employed, a sensation of viscousness, such as accompanies the sound audible in a lung affected with active sanguineous congestion, is perceived. If the quantity of such fluid be increased, the bubbling character is developed; but the complete evolution of the bubbles seems obstructed by the clamminess of the tissue of the sponge.—Again, if a very fine sponge, moderately

* Much difficulty has been experienced in rendering into English the French term *timbre;* Professor Wheatstone objects to the translation *quality,* as not sufficiently definite; but we confess we cannot see why this word may not be conventionally allowed to answer to *timbre,* although it may commonly bear a wider signification. We shall ourselves so employ it, convinced that much less inconvenience will arise from this course than from joining in the habit, so mischievously prevalent at the present day, of interlarding our language with foreign words and phrases. To explain, in a few words, the meaning of *timbre* or *quality,* let us suppose two different instruments—a violin and a flute for example—to sound the *same note* with *the same intensity,* and for *the same length of time;* there will still be a certain peculiarity in each of the notes produced, distinguishing it from the other—that peculiarity is its *timbre.* The physical cause of the special *timbre* of each musical instrument and human voice is still unknown; some attempts at an explanation may be consulted in Müller's Physiology.

soaked with pure water, be compressed at any particular point with the finger, and then allowed to expand, a crepitating sound follows, possessing the precise characters of those puffs of crepitant ronchus, coexisting more particularly with the inspiratory movement, in pneumonia. The perfection of the bubbling character, the duration of the sound, &c., are modified by the manner in which the finger is removed after effecting the pressure. If it be raised in a jerking manner, so as to obstruct the free ingress of air into the tisssue of the sponge, it appears to effect its entry by successive efforts, the bubbling character is incompletely developed, or a few scattered bubbles only are distinguished. Now here we have exactly the characters of the crepitant ronchus of pneumonia, when the entire mass of a lobe is, with the exception of a few vesicles, impermeable to the air; the practical inference from this is, according to M. Fournet, perfectly clear—abundant puffs of crepitant ronchus indicate a more favorable physiological condition of the lung, than a ronchus consisting of a few scattered and ill-formed bubbles. To us it appears that the experiment merely illustrates the practical fact ascertainable by clinical observation. When, of the kind now referred to, the crepitation commonly occurs only at every second or third inspiration, and at the close of the movement, a condition which is known to accompany the period of transition from the first to the second stage of the disease.

M. Fournet further avails himself of his experiments with sponge in illustrating the mode of production of the inspiratory and expiratory murmurs, and their relation to each other. The expansion after compression is accompanied, as we have seen, with a distinct sound; unless a coarse sponge be employed, no appreciable noise is, on the contrary, produced by its compression. The sound elicited in the former instance is analogous to the inspiratory murmur, because resulting from the rush of air into the material experimented on; its *duration* and *intensity* are in the direct ratio of the thickness of the part compressed, in other words, of the quantity of substance acted on. In the same way, according to M. Fournet, in the normal state of the lungs and of the moving powers of those organs—that is, the number of permeable vesicles, and the force of respiration continuing the same—the duration and intensity of the respiratory murmurs will be constant quantities, and may hence be expressed numerically; to this proposition we shall presently have occasion to return. But we may not any longer delay with these experimental enquiries, which are carried much further by the author, and may be varied by any one inclined for such investigation. They certainly illustrate usefully the phenomena of healthy and abnormal respiration; for that the application of these results to the pulmonary tissue is neither strained nor inadmissible seems proved by the fact, that experiments by M. Piorry, on pieces of lung imbibed with various fluids, gave rise to precisely similar sounds. There is excellent exercise for the young auscultator, whose clinical opportunities are limited, with a piece of sponge, a basin of water, and a little gum.

These preliminary notions conduct us to the body of the work, which is divided into eight chapters, embracing enquiries into the physiological sonorous phenomena of respiration; their morbid varieties; the course, relative importance, and combinations of the latter; their classification; the laws regulating their coexistence with inspiration or expiration ; the characters distinguishing both murmurs in the different diseases of the respiratory system ; the primitive seat or place of origin of those sounds; and, finally, the mechanism of their production. To introduce the reader successively to the various details of this comprehensive plan is plainly incompatible with the objects of this Journal : in the selection of topics, we shall principally have in view novelty and practical utility of isolated facts ; though the author would probably object to such a course, as his generalizations and co-ordinations of phenomena exhibit his intellect in the most favorable point of view.

Let us commence then with the normal respiratory sounds in the vesicular section of the apparatus. Nothing is more common than to hear the inspiratory murmur of individuals characterized as "pure and vesicular," when it agrees with the adult type of health; but this expression, if meant to denote the character of the sound, to convey the notion of a successive dilatation of separate vesicles, is seriously erroneous. M. Fournet is perfectly correct in affirming that, if we look for such characters in healthy respiration, we shall search in vain for sound lungs. Far from this, the vesicular character, thus understood, is an abnormal phenomenon; whilst the true healthy sound is a mellow, continuous, gradually developed, breezy murmur, unattended with a sensation either of dryness or humidity. The use of the term vesicular is only justifiable with reference to the presumed seat of the sound. If we add, with our author, that the healthy expiratory murmur possesses precisely the same acoustic constitution as the inspiratory—that it is as soft and breezy, as free from hardness and dryness, and differing therefrom solely in duration and intensity, we shall probably startle no few practised auscultators. At least the testimony of writers of repute is to a different effect. Thus, in the very useful lectures of Dr. Williams, we find it stated that "in expiration there are at most only two kinds of sound, *bronchial* and *tracheal ;*" and on enquiring into the grounds of this affirmation, we learn that it rests, as was to be apprehended, not on direct observation, but on deduction from some ingenious though, we think, fanciful theories respecting the reciprocal influences of the air and pulmonary tissue. "In respiration, the motion begins with the lungs, and the air, passively yielding to it, there is not motion or resistance enough to produce sound, until, by the converging together of the small tubes, the impelled air is gathered into a current in the larger tubes, where, impinging against their sides with its now acquired velocity, it at length produces sound." (p. 33.) Hence, according to Dr. Williams, the expiratory murmur is necessarily, from its seat, bronchial in character. The appreciation of this point is ascribed to MM. Andral, Louis, and Cowan ; now we can affirm that M. Louis neither professes nor ever did profess any such doctrine; and we are persuaded that Dr. Cowan, whose otherwise excellent paper well deserves perusal, has in some instances mistaken the bucco-pharyngeal for the parenchymatous expiratory movement. But the question is neither to be decided by authority, nor by an appeal to *à priori* notions on the physical play of an organ, of the intimate structure of which nothing is in reality known;—it is a question of pure observation. Let the observer place his ear to the chest of healthy subjects, think neither of physics, nor of chemistry, nor of written dogmas, but apply his mind to perceive his own sensations, and he will invariably find that (unless when, as is not unfrequently the case, it is inappreciable by the senses) the expiratory murmur possesses all the softness, gentle breeziness and freedom from bronchial character, which belong to its predecessor in the rhythm of respiration. Above all, he will discover that the period elapsing between the termination of the inspiratory and the commencement of the expiratory sound is an almost indivisible moment; that, consequently, where the one ends the other begins—that if the former be seated in the vesicles, the latter originates there also.

But, as we have already said, these murmurs do differ in two essential points, intensity and duration; the inspiratory exceeds the expiratory in both. M. Fournet has ingeniously endeavoured to give a precise estimate of the degree of difference subsisting between them, by assigning a numerical value to each; and fixes on 10 : 2 as the ratio of their comparative intensity and duration in the healthy state.* Yet, while we applaud the attempt, we must not disguise from our readers the sources of fallacy which interfere with the general truth and applicability of this estimate. In the first place, it is a point of extremest delicacy to determine in any given case how many times longer the first is than the second sound; and skilful auscultators may very readily form different opinions on the point: observers are not agreed as to the proportional duration of the two sounds and period of repose of the heart, yet here the problem is far less delicate. Secondly, does the ratio remain unchanged under all physiological conditions of increase or decrease of the respiratory murmurs, such as those of anhelation, &c.? M. Fournet infers that it does; but whether he has actually observed the fact does not clearly appear: we are disposed, however, judging from a limited number of experiments, to accede to the justness of this proposition, that when the inspiratory sound augments in health, the expiratory undergoes a closely similar proportional increase. Thirdly, is this ratio constant in all healthy subjects, in both sexes and at all ages? Here is the point respecting which we have the strongest misgivings; we might go further, and affirm that it is not—at least as far as regards the murmurs appreciable by the ear. It is in truth utterly impossible, even by causing them to vary their mode of respiring in every possible way, to detect a shadow of expiratory sound in certain subjects; and though the intensity of inspiration is generally low in such individuals, it is clear that the ratio of something to nothing is not as 5 : 1. For the present, however, M. Fournet's estimate may be accepted as a fair approximation to the general truth. Dr. Phillipp writes that no regular proportion prevails, that the expiratory sound may be totally absent, very weak, approaching to, equal to, or exceeding the inspiratory in point of intensity.† This is a most exaggerated statement of the irregularity observable, and evidently proceeds from this writer's having confounded certain diseased states with the healthy condition. The absence of expiratory sound alone cannot lead

* As 5 : 1 would be a more natural way of notating this ratio, that in the text is preferred by the author, as allowing a greater range of figures to express the modifications of increase and decrease occurring in the state of disease.

† Die Lehre von der Erkentniss und Behandlung der Lungen- und Hertzkrankheiten, p. 47.—A useful manual for the author's countrymen.

to any diagnostic error, if the important position laid down by M. Fournet, that such simple absence never depends on an anatomical lesion of the respiratory apparatus, be accurately true; for, under these circumstances, it is a variation not only compatible with but peculiar to the physiological state. This observer, indeed, affirms, that the expiratory sound is never absent alone, unless when patients, imagining that some extraordinary effort is required on their parts to facilitate the examination of their chests, breathe in that convulsive and jerking manner, with which the clinical observer is familiar, and which obstructs materially the circulation of the air in the lungs. To the correctness of this statement we cannot, as may be inferred from the remarks we have just made, by any means accede.

Passing to the section on normal bronchial respiration, we learn that M. Fournet has vainly sought in some individuals for this character, even in the murmurs produced in the interscapular space corresponding to the roots of the large bronchi. A vesicular murmur masks the bronchial in these cases; and the peculiarity is presumed to be due to the investiture of the portion of the tubes in question by a tolerably thick stratum of pulmonary tissue. The author has at least, as he avers, occasionally met with this anatomical disposition, but does not inform us if he has done so in individuals in whom the unusual absence of bronchial sound had previously been detected. We very fully agree with him in considering the region referred to the only one in which bronchial respiration is normally heard; authors have, as we believe, rather speculated than recorded the results of observation in assigning a bronchial character to the murmur in the axillæ and opposite the spine of the scapulæ; and consequently we can by no means assent to the statement made by Dr. Williams, that natural bronchial respiration is heard " over the space of two or three inches on each side of the top of the sternum."

In describing the pharyngeal, buccal, and nasal murmurs, M. Fournet dwells on the error, occasionally committed, of mistaking those sounds for bronchial respiration produced in the region to which the ear or stethoscope is applied. Independently of the difference of *quality* and of the seat of these sounds, which is easily recognized by an attentive and practised ear, there are two modes of correcting the illusion noticed by our author: the doubtful sound, if developed in the pharynx or mouth, may be altered in character by causing the patient to change the form of the openings of those parts, and vary the degree of rapidity with which the air penetrates; and again, as in some instances the pharyngeal sounds are perceived not by the applied but by the free ear, closure of the meatus of the latter will correct the error, as true bronchial or vesicular respiration are never perceived by the distal ear.

The laryngeal inspiratory and expiratory murmurs are, according to this observer, equal in intensity and duration, and may be represented by the number 20. The tendency to equalization of the two sounds increases, the higher the section of the respiratory passages in which they are examined: an observation of very nearly the same kind was made four years since by Dr. Cowan.

The diagnostic value of certain modifications of the respiratory murmurs is found to depend in a great measure on the existence of such modified character in one lung only or in corresponding parts of both organs: in other terms, certain states of respiration derive their sole, others their chief value from comparison, a fact judiciously inculcated by Dr. Stokes. Now, for the employment of this principle with precision, the respective conditions of both lungs in health require to be pre-ascertained; for a very trifling natural difference in the murmurs in each will obviously produce the same effect, as a certain amount of disease would on the supposition of their being naturally identical. Let us suppose, for example, that a practised auscultator detects a delicate shade of difference in the *intensity* of the *inspiratory* murmurs under the clavicles; he will have an important sign of commencing tuberculization on the stronger side, provided, in the healthy state, the intensity is equal on both. Auscultators have carefully examined, therefore, whether the murmurs are similar or dissimilar in the corresponding points of the two sides of the chest; but we regret that the delicacy of the question has already interfered, and is likely to continue to interfere very seriously with uniformity of opinion respecting it. Dr. Stokes dwells on the importance of his discovery, that "in many individuals there is a natural difference between the intensity of the murmur in either lung, and in such cases, with scarcely an exception, the murmur of the *left* is distinctly louder than that of the right lung." * M. Fournet has, on the contrary, satisfied himself that, in persons presenting all the characteristics of healthy lungs, the sounds of inspiration and expiration are precisely identical in all corresponding points: in the few individuals in whom he detected a slightly greater development of the *expiration* under the *right* than the left clavicle, there were some motives for a dubitative opinion respecting the state of the lungs. Not contented with this result of observation, the author indulges himself in detailing some anatomical comparisons between the two lungs, whence it would follow that their physical constitution does not furnish any efficient cause for a difference in their respiratory sounds. This method of investigating the question—a tame imitation of the old system of writers, who busied themselves with ascertaining not what *is*, but what *ought to be*—is obviously not entitled to a particle of confidence. But, admitting the principle of enquiry to be a correct one, the inference to which it leads can hardly be that announced by M. Fournet, for he reasons from an erroneously applied datum respecting the relative size of the two sides of the chest. He states that the transverse diameter of both sides† and the capacity of the summits are equal; but neither these transverse diameters nor the capacity of the summits can really have the influence bestowed on them by M. Fournet; the question of size, as far as it bears on the point at issue, centres in the *general* comparative capacity of the two sides. Now, as has been proved by the researches of M. Woillez, the normal capacity of the right side of the thorax exceeds that of the left by a mean quantity 1·4 centimetres.‡

According to our author, the normal resonance of the voice is somewhat more marked in front than behind, at the summit than at the

* On Diseases of the Chest, p. 394.

† We presume this means when measured internally on the dead subject, though this is not distinctly stated; if the assertion were applied to the external measurement, it would be at direct variance with the statement elsewhere made, that the right transverse diameter is greater than the left.

‡ Vide British and Foreign Medical Review, Vol. VII.

base of the chest, but equal in the corresponding points of both sides. This account in its last particular clashes with the experience of the most eminent men. Often have we heard M. Louis expatiate on the existence of slight natural bronchophony—not as a constant, but as a very frequent condition—opposite the origin of the right bronchus; and we have been too closely familiarized with the exquisite perfection of M. Louis's acoustic faculty to question his accuracy, more especially as his opinion harmonizes with the experience of Sir James Clark and Dr. Stokes. The former observer recognizes greater resonance under the right than the left clavicle as a condition of health; the latter incidentally mentions his belief, that a similar superiority prevails over the entire of the right side of the thorax. (Op. cit., p. 497.)

M. Fournet considers the natural resonance of the voice much more marked in persons with a grave than an acute voice. Dr. Williams maintains on the contrary that it is most distinct when the voice is high or treble. The French writer's opinion is contradictory of an admission elsewhere made, that bronchophony and pectoriloquy have little symptomatic value in children, precisely because the natural resonance is in them very generally so intense as to simulate those morbid conditions.

In his division of the *morbid* phenomena of respiration, our author, like some of his predecessors, distinguishes transformations of the normal murmurs from another order of sounds, which are wholly new formations. The diseased changes to which the natural murmurs are liable are all referred to the heads of *augmentation*, *diminution, cessation* and *perversion.* Those belonging to the last class form a sort of transition between the modification and the new production: thus the metallic tinkling of hydropneumothorax belongs to the latter class, though it also follows in the train of alterations of *quality* belonging to the former. The author's experiments on sponge mark the error of Laennec in supposing that the various ronchi are not modified conditions of the normal respiratory murmurs; they exemplify the gradual transition from the natural to the bubbling character of ronchi. M. Fournet displays, at the same time, his usual ignorance of the literature of this and indeed of his own country, in fancying himself the first to proclaim the falsity of Laennec's belief.

The normal intensity and duration of the inspiratory sound being represented by 10, the extreme degrees of increase and decrease mark 20 and 0; between the maximum point of elevation and that of total cessation, all intermediate grades are observed. A remarkable difference in the mode of production of increase and diminution is, according to M. Fournet, that the former change never springs directly from any physical alteration in the pulmonary structure, and is produced, not in diseased parts, but in circumjacent healthy tissue; in a word, it announces the general fact, that a part of the lung supplies, by increased action, the functional incapacity of another, and characterizes *supplementary* respiration. On the contrary, the diminution of the murmur is the direct effect of some physical obstruction to the entry of the air, and represents the intensity of that obstruction. The importance of this modification, which, in the great majority of cases, affects both the intensity and duration of the sound, is apparent from the fact, that there is scarcely an organic disease of the larynx, trachea, bronchi, pulmonary tissue, and pleura, which, as well as certain spasmodic affections, is not productive of it to a greater or less amount.

In speaking of the total cessation of the respiratory murmurs, occurring in well-marked pneumothorax, in cases of abundant liquid effusion into the pleura, &c., M. Fournet states, that towards the end of the inspiratory movement a gentle sound may occasionally be perceived, which appears to result from the lateral pressure of the condensed pulmonary tissue, by the air which is prevented from entering the bronchial ramifications. He conceives that the phenomenon thus arising may be rendered tolerably intelligible by the term *sound of pulmonary compression;* but, we confess, it conveys to us no very distinct idea. Dr. Williams, who long since noticed the peculiarity in question, described it more happily, as an abrupt stoppage of inspiration, accompanied with a kind of *hic.*

In health, the inspiratory sound is uniform and continuous; this condition constitutes, according to M. Fournet, its normal *rhythm.* In cases of sharp pleurodynia, he states, this rhythm changes; the murmur becomes abrupt, jerking, and divides into several successive and unequal parts. In incipient pleurisy, in the dry stage, a similar state is however observed; so that this observation throws no new light on the diagnosis of these two complaints. During the alteration of inspiratory rhythm, the expiratory remains unchanged; a fact easily intelligible.

A few points in the remarks on alterations of *quality* of the inspiratory murmur deserve notice. These changes are arranged according to their rate of intensity, in the following ascending scale: 1, the clear character; 2, the ringing; 3, the blowing; 4, the bronchial; 5, the cavernous; 6, the amphoric; 7, metallic tinkling. M. Fournet takes much credit to himself for seizing the intimate relation of these successive gradations, instead of regarding them, with Laennec, as distinct and isolated phenomena:—with what real claims to originality he plumes himself on this specimen of his acuteness, we need not remind the reader. He generalizes the anatomical conditions giving rise to alterations of *quality*, referring them all to solidification of the pulmonary tissue, increased size of the cavities in which the air circulates, or both lesions combined. We are scarcely satisfied that M. Fournet has not been over minute in attempting to establish those intermediate degrees of change between the natural and bronchial *qualities;* and it is certain, from his own admission, that these tranformations cannot be detected in acute solidification, whatever may be the case when that process is chronic. In pneumonia and pleurisy, for example, the *clear quality* and the *ringing*, which is a slightly increased degree of the former, are avowedly not detected in the transition to bronchial respiration; M. Fournet attributes this to the sudden and rapid increase of density undergone by the pulmonary tissue,—with what justness, further experience must decide.

Change of *quality* invariably, according to our author, commences with the expiratory murmur, and, after a period of variable duration, becomes manifest in inspiration. In its disappearance, it follows the opposite order; expiration is the first affected and the last released. The morbid *quality* may pass to the fourth grade in expiration, without having exhibited itself, even in the lowest degree, in the inspiratory murmur. This is a generalization of the point established by Jackson, in respect of the expiration in incipient tuberculous cases. Hence the coexistence of even the slightest degree of altered *quality* with the inspiratory sound, announces a more advanced state of disease than even the fourth degree of change in expiration: this and allied facts, should they be found of uniform occurrence, may prove of considerable service to the practitioner in ascertaining the active or quiescent state, locally considered, of tuberculous disease. In their slightest degrees, the morbid changes under consideration appear only at the close of the inspiratory and expiratory murmurs; as the modification rises in the scale of intensity, it affects an increasing share of each sound.

The expiratory murmur is subject to much greater increase in point of intensity and duration than the inspiratory: if we credit M. Fournet, the maximum increase in these respects may be represented by the number 20, that already employed to designate the corresponding condition of the inspiratory sound. Now, as in the normal state, the former and the latter murmurs are made respectively equal to 2 and 10, it follows that while inspiration is only capable of acquiring double its healthy duration, expiration may attain ten times the natural proportion. And again, as it is elsewhere stated, that while the expiration undergoes this enormous rise, the inspiratory sound may fall to 1, it follows that instead of the expiration being only one fifth as intense as the inspiration, it may be twenty times as intense as the latter; and hence that it may actually bear one hundred times a higher proportion to the inspiratory murmur than natural. We are almost persuaded there is exaggeration in this expiratory estimate; at least we have never, ourselves, observed a degree of prolongation in cases of vesicular emphysema (wherein the abnormal extension has to us appeared to reach its utmost limit) which could be rated at more than five or six times the natural amount.

Augmented expiration may either coexist with a proportional increase in the inspiratory murmur, or the healthy ratio of the two phenomena may be destroyed by an accompanying fall in the inspiration. The former condition occurs in puerile or supplementary respiration; the latter in the early stage of phthisis, and in emphysema: these are indeed the only affections in which the disproportion exists to a very large amount, and hence its special value in their diagnosis.

There are two circumstances likely to induce a false estimate of the degree of abnormal prolongation. With one of these sources of fallacy, the bucco-pharyngeal murmur, we have already made the reader acquainted, and stated M. Fournet's instructions for correcting the illusion. The other originates in the fact, that the inspiratory murmur sometimes diminishes in length, while the expiratory remains stationary: if the ear take cognizance simply of their *proportional* condition, the error of supposing the duration of the expiration increased, will be very readily committed; to correct it, the attention must be directed to the *absolute* length of each murmur. The former circumstance is by far the most practically important, and we have had sufficient personal acquaintance with its deceptive influence, to recommend the young auscultator to familiarize himself thoroughly with the characters of the buccal, nasal, and pharyngeal sounds, and the means recommended by M. Fournet for correcting the erroneous impressions to which they are prone to give rise.

Diminution of the expiratory sound is much more rarely noticed than of the inspiratory; the latter often falls to 4 or 5, while the former retains its normal value, 2. But if the cause of the depression of the inspiratory murmur continue in action beyond this point—in cases of pleuritic effusion, for instance,—the expiratory sound gradually falls to 0. In respect of the relation subsisting between the two murmurs in the morbid state, our author asserts the accuracy of the following general propositions. All affections which produce a simultaneous and proportional decrease in the intensity and duration of inspiration, are attended with diminished expiration; for example, pneumonia, pleurisy, pulmonary œdema, and apoplexy. On the other hand, when the intensity and duration of inspiration undergo opposite modifications, the one of increase, the other of diminution, the expiratory murmur is augmented; of this fact, pulmonary emphysema and tuberculization supply illustrations.

A parallel instituted between the morbid variations of the two murmurs, discloses a curious opposition in the laws regulating them; a few of these may be stated, as indicating the general character of the whole. The intensity and duration of the expiratory sound almost always rise or fall coevally; these characters frequently undergo opposite changes in inspiration. Modifications of *quality* belong more especially to expiration; those affecting the properties of dryness and softness, to inspiration. Diminution, affecting both murmurs, appears always first, and advances to its extreme degree more rapidly in inspiration than in expiration; in the return to the healthy standard the expiratory, on the contrary, precedes the inspiratory sound. In the particular facts related regarding each murmur, we have furnished the reader with materials for extending the parallel.

The exaggerated importance attached by Laennec to the distinction of morbid vocal resonance, to pectoriloquy and ægophony, for instance, as signs of excavation in the substance of the lung, and of the presence of a thin stratum of liquid in the pleura, is matter of daily demonstration. M. Fournet, who with even greater boldness than his predecessors affirms that it is in some cases impossible to determine whether a given ringing of the voice be ægophony, bronchophony, or pectoriloquy, has judiciously arranged the arguments proving the comparative futility of their distinction. We recommend this chapter to that rather numerous class of practitioners, who somewhat perversely cling to the correctness of Laennec's doctrine,—to persons who fancy themselves justified in invariably distinguishing hepatization from effusion, by the absence or presence of an ægophonic twang in the vocal resonance, or predicate the existence or non-existence of caverns from that of pectoriloquy. But while we express ourselves thus strongly against the habit of trusting mainly to these signs, more especially to pectoriloquy, we by no means deny their occasional value, and dispute not their general importance as allied signs. The accurate information derived from certain forms of pectoriloquy, has been satisfactorily pointed out by Dr. Williams; and, as M. Fournet observes, the strange, mysterious, ventriloquous sound produced in caverns, when the laryngeal voice is reduced by the progress of the tuberculous disease to a mere whisper, can hardly be confounded with any other known phenomenon.

The augmentation of natural adult respiration, termed *puerile* by Laennec, because resembling that of children; *supplementary* by Andral, from its making up for diminished energy in some other part of the lungs; undergoes, upon inadequate motives, a second change of title in M. Fournet's volume: the idea of the new epithet, *exaggerated*, is contained in Andral's term, which, besides, marks the most important signification, practically speaking, of such respiration. The chief character of this form of respiration is an increase of intensity and duration of both murmurs, proportionally greater in expiration, and accompanied with a trace of *clear* or *blowing quality*. The comparatively greater increase of the second murmur, and the *quality* of both sounds in supplementary respiration, make it difficult to distinguish this form from cases in which the expiratory murmur has singly undergone a *morbid* increase. This difficulty, as we shall hereafter show, arises in the first stage of phthisis. In emphysema it may also be encountered, and the distinction is hardly to be made without the aid of other signs, e. g. hardness and dryness, in the murmurs. In truth it seems highly probable that, as M. Fournet hints, supplementary respiration, and the state characterized by prolonged expiration, are one and the same, fundamentally, and acquire their distinctive peculiarities from the combination of other changes with the latter.

Supplementary respiration is sometimes of no mean importance as a diagnostic sign; of this fact, strikingly remarkable in certain cases of pneumonia, M. Fournet gives a useful illustration. In three individuals expectorating the rusty viscid sputa, and exhibiting the general symptoms of pneumonia, the ordinary physical signs could not be detected. The only unusual phenomenon was marked supplementary respiration on one side of the chest; in two of these cases the ordinary signs subsequently appeared in the situation where the augmented respiration had existed; in the third, the latter phenomenon disappeared with the rusty sputa and general symptoms. Here were indubitable examples of central inflammation; in the latter instance, persisting to the end in its original situation; in the two former, spreading to the periphery of the lung. In cases where the characteristic sputa are wanting, as well as the usual physical signs,—an exceedingly rare coincidence however,—M. Fournet's observation may, we doubt not, be found of utility. Allied with this subject is the important question of the earliest signs of pneumonic inflammation, upon which a few words here will not be misplaced. Dr. Stokes, as our readers are aware, teaches the existence of a distinct stage of pneumonia prior to that of engorgement, and is led to believe that "an intense puerility of respiration in the affected part" will be found to accompany the earliest seizure of the pulmonary tissue, and hence point out the precise spot in cases of spreading inflammation about to become the seat of the crepitant ronchus. Dr. Williams, in imitation of Dr. Stokes, is of opinion that "the natural respiratory murmur will be rendered rough, and perhaps sharper, before the crepitation begins." But on the other hand, M. Grisolle, an observer of respectable Parisian reputation, declares "he has been enabled to follow the gradual extension of the inflammation, by the *loss of force and purity* on the part of the respiratory murmur, in a circumscribed point adjoining the manifestly inflamed part."* Now the particulars above related, from M. Fournet's work, testify to the accuracy of Dr. Stokes's *obser-*

* Mémoire sur la Pneumonie, p. 11. Paris, 1836.

*vation* respecting the sign of imminent attack of a particular part by the inflammatory action: but there is this fundamental difference between the two observers, that the one considers the puerile character produced in a still healthy part, the other in tissue in an incipient state of inflammation; in the one case it announces the actual existence of adjacent engorgement, in the other it implies that engorgement is ready to occur. That Dr. Stokes's *rationale* is probably (judging from close analogy, as well as from the third case alluded to by M. Fournet,) erroneous may, we think, be assumed; and, as we shall presently see, M. Fournet's experience of the signs produced in actually congested tissue, tallies more closely with that of M. Grisolle than of our countryman.

Under the name of *pulmonary crumpling sound* (bruit de froissement pulmonaire), the author describes a phenomenon apparently occurring, as he alleges, in the substance of the lung, and differing from all auscultatory signs hitherto noticed. In its most intense form, this sound very closely resembles the *new-leather creak of pericarditis*, differing therefrom solely in its greater acuteness of *quality;* in a less marked degree it constitutes a sort of plaintive noise, varying in tone with the rapidity of respiration; and, in the weakest and most common form, bears a close similitude to the gentle, quick, dry sound, elicited by blowing on fine paper. This phenomenon is generally produced in a very confined space, ordinarily coexists with inspiration only, and has been noticed by M. Fournet in the first stage of phthisis, in one case of encephaloid cancer of the mediastinum, and in another of non-tuberculous cavity of the summit of the lung. Some phthisical subjects experience a feeling of discomfort in the situation where the sound is audible; but this is rare. The anatomical conditions under which it arises are stated to be, a mechanical obstacle to the expansion of the lung, lobular induration of the pulmonary texture, and alternate flapping backwards and forwards of a fibrous lamina forming the wall of a cavern. As a sign of intra-thoracic tumour it possesses no value; it is of greater utility in cases of phthisis, as it existed in one eighth of tuberculous subjects attentively examined.

We shall recur to the subject of this sound, when engaged with the early diagnosis of phthisis. Meanwhile we may venture to express our doubts, founded very much on his own description, of M. Fournet's correctness regarding the location, mode of production, and close relationship of these modifications of sound. We can discover no proof of their being connected in the manner alleged; there is no attempt even made to point out the stage of transition from one to another, and that a sound like the pericarditic leather creak should be produced by the mere compression of indurated pulmonary tissue by the inspiratory rush of air into the neighbouring vesicles is, primâ facie, most questionable. It is very probable that the most delicate form of *crumpling sound* may be thus developed; but the intense variety referred to appears to us an uncommon and peculiar species of friction sound, produced in pleural false membrane, or similar structure. In the case of cavern adverted to, such was confessedly its mode of production. We have before us notes of a case of chronic pleurisy in a tuberculous subject, observed in August, 1835, in which we find these words: "Under the left clavicle the inspiration is masked by a well-marked *friction* sound, which does not exist in expiration, though when the patient breathes expansively, there is a well-marked expiratory murmur. In the supra-spinata fossa a sound resembling the creaking of new leather coexists with inspiration and expiration; opposite the spine of the scapula this changes into a slight crackling sound, still masking the respiratory murmurs; these are heard about and within the angle of the scapula, the inspiratory sound is feeble, unaccompanied with ronchus, the expiratory prolonged and blowing." Now let us contrast the characters assigned to the creaking and friction sounds:

| *Creaking variety of crumpling sound,* | *Friction sound,* |
|---|---|
| Coexists with inspiration only. | Almost always coexists with both movements. |
| Is continuous, and unaccompanied with any sensation of displacement. | Is jerking, and attended with a sensation of elevation and depression. |
| Is most frequently heard at the summit of the lungs. | Is most commonly met with at the central part of the posterior surface. |
| Does not produce any sensation appreciable by the patient himself. | Is not unusually felt by the patients themselves. |
| Is accompanied with no thoracic fremitus. | Gives rise to distinct fremitus. |

The reader will observe, that the phenomenon under the clavicle possessed the majority of the characters of the crumpling sound, while the sound in the back was that of friction. Now the similarity in their characters appears presumptive evidence of the similarity of the tissue in which they are produced, and all the differences noted between them may be accounted for on the supposition, that the one arises from the unfolding of indurated pleural tissue, while the other originates in the collision of granulations, or laminar false membrane. It must be remembered, too, that although the distinctive peculiarities of the friction and crumpling sounds generally exist as they are stated above, yet each character may be absent, or those represented as peculiar to the one coexist with the other. The subject requires further examination.

Under the title of *humid ronchus with continuous bubbles,* M. Fournet describes a morbid sound which, he states, existed in twenty-three subjects, the only ones carefully examined, affected with active sanguineous congestion of the lungs. This is a vesicular ronchus, composed of bubbles with a peculiar character of viscidity and humidity, and which, in the language of our author, instead of attaining a completely spherical shape before bursting, form a third or a half only of a sphere,—an imperfection of development very distinctly traceable to the viscous quality of the liquid forming them. They hold a mean rank, in point of size, between those of the crepitant ronchus of pneumonia and of mucous ronchus. They are few in number, three or four only occurring during each inspiration (with which movement they coexist exclusively), and are slowly formed. Each new bubble is produced before the full development and explosion of its predecessor; hence the sensation of continuousness with which this ronchus is accompanied.

We have closely reproduced the more striking characteristics of this ronchus, as detailed by M. Fournet; the minuteness of analysis gives it an apparently novel character, but we have no doubt it has been observed by many, and confounded with the subcrepitant ronchus of acute capillary bronchitis. The latter is however, according to the author, distinguishable from it by the larger size, greater number, more perfect sphericity, and comparatively rapid production of its bubbles, from its existing over a wider space, and at the posterior bases of *both* lungs, while the ronchus of active sanguineous congestion is usually confined to one side.

In the normal state, the movements of expansion and retraction of the lungs do not give rise to any appreciable sound, having its seat in the pleuræ. Of this fact, ascertainable by auscultating healthy individuals, and demonstrated on horses by M. Andral, the author adduces new proof from the examination of the thoracic and pulmonary movements in *two* rabbits. These experiments would not have been worth notice, were they not cited as proving—for M. Fournet affirms that M. Piorry and himself *saw* this through the flayed walls of the animals' chests—that friction of the pleural surfaces against each other regularly occurs in the healthy state, during inspiration and expiration. The observation clashes with some physical principles, and the nice deductions of M. Woillez on the reciprocal influence of the thorax and its contents; but as we have not space to enter on a consideration of the subject, we must content ourselves with referring the reader to our review of that gentleman's recent work. Were M. Fournet's experiments more numerous, and of unimpeachable accuracy, no à priori consideration should interfere with the inference they furnish; but they are few in number, and (as all who have examined the curious question of the effects of penetrating wounds on the motions of the lungs will at once recognize on perusal of their description) performed by a person most imperfectly acquainted with the complex nature of the phenomena he was about to analyze.

The morbid phenomena originating in collision of the pleural surfaces are examined with characteristic minuteness. Three varieties of friction sound are here admitted: 1, *The grazing sound* (bruit de frôlement); 2, *Friction sound properly so called* (bruit de frottement); 3, *Grating sound* (bruit de râclement). They are always audible in inspiration, not appearing in expiration unless strongly marked; thus the *grazing* variety is not perceived in the latter movement, while the others manifest themselves in both; under all circumstances they appear first in, and disappear last from, inspiration. Their occurrence over an extensive surface is of favorable augury, as such state usually announces rapid and uniform absorption of pleuritic effusion.

M. Fournet's experience confirms the conclusion already arrived at by Andral, Louis, Dr. Stokes, and others, respecting the non-existence of friction signs in interlobular emphysema. Indeed, the error of Laennec, in assigning this causation to the sounds in question, is now generally recognized.

The rarity of friction signs, compared with the frequency of pleuritis, has not failed to strike our author. Upon the more familiar causes of this disparity it is needless to dwell; we may however notice that M. Fournet considers diminished facility of locomotion in the lung, either from general pleuritic adhesion or extensive engorgement of its tissue, as capable of preventing their development. That they do cease, under the circumstances, had already been noticed by Dr. Stokes, who tendered, as an hypothesis, the explanation now announced as matter of certainty. Indeed, the fact that the supervention of friction sounds, in some cases of pleuro-pneumonia is coeval with the period of resolution, not of the pleuritic effusion, but of the coexisting pneumonia, seems otherwise inexplicable.

According to M. Fournet, some trace of respiratory murmur always coexists with friction signs, because the expansion of the lung, and the rubbing which follows, cannot be effected without the penetration of some air into the vesicles. We are not unwilling to admit the accuracy of the latter clause, but question the truth of the inference from it, and deny the propriety of the mode in which that inference is drawn. Dr. Stokes was, we believe, the first to point out the interesting fact that friction phenomena may coexist with "great and universal dulness," and consequently with extensive liquid effusion; now, though Dr. Stokes does not mention precisely the condition of the respiratory murmur under these circumstances, yet if the dulness were at once great and universal, that these were absent seems the only reasonable conclusion. Nevertheless, it may be urged in favour of M. Fournet's opinion that, as in a case of abundant effusion attended with friction sound, he traced their production to some membranous adhesions retaining one part of the lung in rather close connexion with the costal pleura, the respiratory murmurs must have been audible opposite those adhesions, and are likely to have been similarly perceptible in Dr. Stokes's cases.

We can hardly assent to the correctness of another of our author's "laws,"—that the duration and intensity of the respiratory murmur generally decrease in proportion as the friction sounds reach a higher type. The occasional coexistence of the most intense form of friction sound (*grating variety*) with minute granular elevations, either on the surface of a false membrane or of the pleura itself, a circumstance adverted to by himself, is clearly at variance with the supposition of proportional diminution of the respiratory murmurs.

The grazing variety differs, in some important particulars, from allied phenomena. Though remarkable for mobility, it occurs more frequently at the upper part of the pleura than elsewhere; the changeableness of its situation depends on its being produced by local and transitory attacks of pleurisy, themselves determined by eccentric tuberculization of the subjacent lung. It is ordinarily of short duration; a single day suffices for its production, development, and termination, and this series of phases may, as M. Fournet has observed, be accomplished several times successively, in the course of a few days. Should these statements be verified, they must give a new character of precision to the diagnosis of local pleurisy, and perhaps help to determine the still unsettled question of the cause of the wandering thoracic pains of phthisis.

On the causes of the variable duration of friction signs, nothing novel is related. M. Fournet however states, and the statement is to us new, that a blister, applied in the situation where the rubbing sound is audible, hastens its disappearance. We are unfortunately left in the dark as to the number of times the fact was observed; but, admitting the occurrence to be a common one, how is the effect produced? "In one case, two blisters were applied successively; on both occasions the friction sound disappeared during the period of suppuration, and reappeared when they were suffered to dry up." We confess we cannot comprehend these changes, unless by supposing that the blisters produced slight effusion in the subjacent portion of pleura, and so deprived the false membrane of the property of dryness essential to the development of the sounds in question. Dr. Macartney was in the habit of remarking, in his Lectures, that the cutaneous irritation produced by *flogging*, often brings on pleurisy; now we should not wish to draw any decisive inference from the analogy here presented, even were the alleged fact clearly proved, but, coupled with the phenomenon detected by auscultation, it rather warrants doubts, which are justified upon other scores too, of the real *therapeutical* power of blisters *applied over the affected part*, in pleurisy. We are the more anxious to draw attention to this point, as young practitioners, deceived by the marked relief of pain afforded by the application of blisters, imagine that in prescribing these epispastics they are really curing the disease, and are hence not unlikely to neglect means of less contestable efficacy. By *therapeutic* power, we mean that of diminishing the mortality, or shortening the mean duration of a disease.

The sudden disappearance of friction signs, originally developed on the absorption of an effusion, announces the return of the latter, or as a corollary from the proposition already stated, the supervention of pneumonia; M. Fournet affirms that in one instance this change in the pleural signs first awakened his suspicions, confirmed by the event, of the latter disease having appeared. There are several other particulars in this section well worthy of notice; but we must allow the reader to discover them for himself.

In his examination of *vesicular* ronchi, M. Fournet confirms the general observation, particularly dwelt on by Dr. Stokes, respecting the occasional absence of crepitant ronchus, both previous to pneumonic solidification and after its resolution. While upon this point, we may take the opportunity of stating our belief that Dr. Stokes has rather undervalued this ronchus as a sign of pneumonia. The coarse auscultator, to whose ill-educated sense all crepitating murmurs appear *the* crepitant ronchus, will no doubt sin frequently in his judgment respecting the presence of pneumonia, when no such affection really exists, if he presume to found his diagnosis on his acoustic perceptions alone. But of the *pathognomonic* character of the crepitant ronchus, formed of minutely delicate, dry, uniform, closely-pressed, regularly-rounded bubbles, bursting as with a sudden puff under the ear, we, with M. Fournet, entertain not a shadow of doubt.

The distinction of the second and third stages of pneumonia has long, confessedly, been one of the most puzzling points of semeiology. M. Bouillaud has, it is true, lately written, apparently with the view of enhancing the excellence of his marvellous *saignées coup sur coup,* as if the distinction were to him matter of facility; but he has convinced none, even of his countrymen, of his prowess, except those whose natural vocation it is to bow the head when "an authority" opes his lips. Dr. Stokes, however, some time since, announced his persuasion that the supervention of the third stage (that of interstitial suppuration) might be detected, with tolerable certainty, by the coexistence of "a sharp and peculiar muco-crepitant ronchus with bronchial respiration;" and M. Fournet, believing himself, as usual, original, confirms the accuracy of this statement. He adds the important observation, that the ronchus coexisted with inspiration only: we call this important, because it shows that the phenomenon was not produced in the bronchi, and is therefore not the same as that which Laennec, à priori, as it is suspected, assigned as characteristic of pulmonary suppuration.

In his fourth chapter the author conveys, in a tabular form, the substance of his remarks on the healthy and morbid characters of respiration, and classifies the various ronchi. The presumed seat of the phenomenon forms the main groundwork of his classification; the character of dryness or humidity gives rise to the formation of sub-classes. The idea of this arrangement is far from new, but is here carried out more completely than elsewhere. In respect of their seat, they are divided into six classes,—intra-vesicular, extra-vesicular, bronchial, tracheal, laryngeal, and bucco-pharyngeal. The intra-vesicular class contains five species,—the humid crepitant, with continuous bubbles, already described at length; the sub-crepitant of pulmonary œdema, the sub-crepitant of acute capillary bronchitis, the ronchus crepitans redux of pneumonia, and the primary crepitation of the same disease. Under the head extra-vesicular, are included those morbid sounds, presumed to originate in the actual texture or parenchyma of the organ. The term is evidently ill-judged, for it is plain that tracheal ronchi have quite as fair a claim to such title as those on which our author confers it. Be this as it may, the class contains two subclasses, founded on the fact of the organ having undergone excavation, or being free from solution of continuity. To the latter belong the pulmonary crumpling sound, and the dry crackling ronchus (of which we shall presently give a full description), produced, as M. Fournet imagines, by the crumpling of the pulmonary tissue against tuberculous deposits. With the former, rank the humid crackling ronchus, the clear mucous, or cavernulous of Hirtz, the dry cavernous, and the humid cavernous, or gurgling.

With the remaining classes we find no reason to detain the reader; and pass on to the chapter developing the laws of coexistence of the morbid phenomena of respiration with the inspiratory and expiratory sounds respectively. The separate appreciation of the two sounds, under all conditions of disease, has led M. Fournet to the conclusion, that such laws do prevail, and that intimate acquaintance with them frequently furnishes a valuable guide in practice. Of this we have, indeed, already given one rather striking illustration. We have shown too, that, according to our author, the progress of certain affections may be traced by the manifestation of a morbid *quality* in both sounds, which had previously accompanied one only. Of the constancy of these "laws," we are of course, for the present, obliged to accept M. Fournet's assurance. The greater number of morbid characters belong to inspiration; but those of highest diagnostic importance appear in expiration; none are peculiar to the latter murmur only. The laws of coexistence may be traced to a certain number of general principles, of which the following are the most striking and important. 1. The closer the proximity of the seat of any ronchus to the pulmonary vesicles, the more marked is the tendency to exclusive coexistence with inspiration. 2. On the contrary, the further the seat of any ronchus is from the vesicles, the more uniformly does its production coincide with both movements. As a corollary from these two propositions it follows, that the proneness to exclusive coexistence with inspiration increases in the inverse ratio of the caliber of the tubes in which the ronchus originates; and this holds good in respect of the ronchi of cavities of new formation and of increasing size,—witness the successive series of the humid crackling, the cavernulous and cavernous ronchi. 3. The larger the bubbles of humid ronchi, the more frequently do these coexist with both movements, and vice versâ. 4. The greater the intensity of dry ronchi, the more commonly do they affect both murmurs; the weaker they are, the more exclusively do these appear in inspiration only: as an instance of this, dry bronchial ronchi may be compared with the crumpling and dry crackling sounds of phthisis. 5. Ronchi of grave tone coexist more especially with expiration; those of acute tone, with inspiration. 6. Ronchi which are exclusively inspiratory appertain, in general, to more serious diseases than those audible with both movements; the humid crackling and humid cavernous ronchi form the only exceptions to this rule.

In the following table of the author's we present our readers with the means of increasing the list of general principles commenced above, and of more accurately estimating the originality and character of M. Fournet's researches on this section of his subject, than we could even by the fullest commentary.

TABLE, *showing the mode of coexistence of the Morbid Phenomena of Respiration with Inspiration and Expiration.*

[The order in which the different phenomena are set down in each division, exhibits the degree to which they relatively acknowledge the law regulating them all.]

A. *Morbid Characters coexisting exclusively, or almost exclusively, with inspiration.*

1. Humid ronchus with continuous bubbles.
2. Primary crepitant ronchus of pneumonia.
3. Mucous ronchus of third stage of pneumonia.
4. Grazing pleuritic sound.
5. Pulmonary crumpling sound.
6. Dry crackling ronchus.
7. Subcrepitant ronchus of œdema.
8. Subcrepitant do. of capillary bronchitis.
9. Ronchus crepitans redux of pneumonia.

N.B. The first three sounds coexist exclusively with inspiration; the others sometimes occur in expiration also, but exceptionally only. The frequency of these exceptions increases from No. 4, downwards.

B. *Morbid Characters coexisting with both movements.*

1. Humid cavernous ronchus.
2. Dry cavernous ronchus.
3. Dry { Bronchial / Tracheal / &c. } ronchi.
4. Cavernulous ronchus.
5. Grating pleuritic sound.
6. Friction pleuritic sound.
7. Augmentations of intensity.
8. Diminutions of intensity.
9. Augmentations of duration.
10. Diminutions of duration.
11. Amphoric character.
12. { Cavernous character. / Veiled puff.
13. Bronchial character.
14. Metallic tinkling and echo.*
15. Blowing character.

* In some cases of hydropneumothorax with perforation, observes M. Fournet, "the *metallic tinkling* of Laennec is not to be discovered; but, instead, the amphoric character of the respiration seems to reverberate in a sort of vague diffused echo, which rings like the voice under an archway; this phenomenon may be called *résonnance métallique*; it often accompanies the voice and cough." The English reader will here recognize the precise description, almost the very words of Dr. Williams, in reference to the phenomenon of *tinkling echo.*

16. Ringing character.
17. Clear ditto.
18. Mucous ronchus.
19. Humid crackling ditto.
20. Dry, hard, rough, laborious character.
21. Humid character.

C. *Morbid Characters coexisting chiefly with Inspiration.*

1. Diminution of duration and intensity.
2. Complete cessation.
3. Humid character.
4. Dry character.
5. All varieties of friction sound.
6. Pulmonary crumpling sound.
7. Dry crackling ronchus.
8. Subcrepitant ronchus of œdema.
9. Subcrepitant ditto of capillary bronchitis.
10. Ronchus crepitans redux.
11. Humid crackling ronchus.
12. Buccopharyngeal ronchi.
13. Cavernulous ronchus.
14. Gurgling ditto.
15. Humid, bronchial, tracheal, laryngeal ronchi.
16. Dry acute-toned bronchial, cavernous, tracheal, laryngeal ronchi.

D. *Morbid Characters coexisting chiefly with Expiration.*

1. Augmentation of intensity and duration.
2. Metallic tinkling and echo.
3. Clear character.
4. Ringing ditto.
5. Blowing ditto.
6. Bronchial character.
7. Cavernous ditto.
8. Amphoric ditto.
9. Dry grave-toned bronchial, cavernous, tracheal, laryngeal ronchi.

E. *Morbid Characters coexisting first with Inspiration, and then extending to Expiration.*

1. Pleuritic friction sounds.
2. Dry and humid crackling ronchi.
3. Hard, rough, dry, laborious character.
4. Humid character.
5. Crepitant ronchi, primary and redux.

F. *Morbid Characters coexisting at first with Expiration, and then extending to Inspiration.*

1. Clear character.
2. Ringing ditto.
3. Blowing ditto.
4. Bronchial character.
5. Cavernous ditto.

In confirmation of the utility of the distinction of ronchi, &c., into inspiratory and expiratory, and those common to both movements, our author collects together some of the most striking examples of differential diagnosis obtainable thereby. We have already, however, touched on the majority of these, and shall only notice two more points. There is no small difficulty, we have said, in distinguishing supplementary respiration from respiration with the expiratory murmur morbidly prolonged; now, if change of *quality* of the bronchial type exist in expiration only, all hesitation is at an end—the case is certainly not one of supplementary respiration. But important as this distinction is, scientifically, in all cases, and practically in many, there are circumstances in which it may have little value in the latter point of view. Let us suppose a respiration of the doubtful character alluded to, audible under the clavicle: to whichever species it really belongs, it may announce the presence of tubercles; if it be supplementary, we conclude that the foreign bodies are rather deeply seated in the lung; if of the other species, that they are close to the periphery: the practical man will hardly thank us for the delicate distinction.

If M. Fournet be correct, the primary crepitant ronchus coexists with inspiration only; the ronchus redux with inspiration chiefly, but slightly with expiration: the inference is, that the period of the disease may thus be ascertained in a patient observed for the first time, and under unfavorable circumstances for learning the previous course of the complaint. But the observations of two distinguished home auscultators do not coincide with those of M. Fournet. Thus Dr. Williams, speaking of the primary ronchus, states that " it is first heard at the commencement of inspiration and the end of expiration; but it soon accompanies the whole respiratory act, and, in advanced degrees of the first stage, is heard only at the end of inspiration and the beginning of expiration."* And Dr. Stokes, describing the ronchus redux, affirms that " it is to be heard during the whole inspiration, and, in a diminished degree, during expiration;" and, so far agreeing with the French observer, adds, that " in other cases the first part of the inspiration is pure, and the râle only appears at the termination," and has once observed " râle first, and then pure vesicular murmur." The impetus which the promulgation of M. Fournet's doctrines will, we are convinced, give to the study of the two murmurs, must soon settle questions of this character; and it is on this account we abstain from noticing numerous points in the table which our own experience would lead us to regard as of doubtful accuracy. We would, however, distinctly state that in all probability the characters or phenomena placed lowest in each division very frequently infringe the law to which they are referred.

A brief examination of the physical signs of the different affections of the lungs follows. Here there is little original matter, except the careful statement of the respective conditions of the two murmurs; to these we have already, as far as is practicable, referred. The exposition of the signs of emphysema is rendered curious by the author's fancying himself the first to study the movements of the chest in that affection with precision.† He has, however, fairer claims to the character of an original observer, in respect of a disease termed active congestion of the lungs; and as both symptomatically and anatomically this affection probably possesses some importance, we shall condense the more essential particulars related about it. If, as M. Fournet conceives, it be really the preparatory stage of pneumonia, and by early recognition of it the development of the latter severe disease may occasionally at least be prevented, it needs no stronger claim to the notice of the English practitioner.

This affection, which is described from the observation of twenty-three cases, chiefly occurred in subjects between the ages of seventeen and twenty-five; was apparently little influenced by sex; most commonly attacked muscular subjects of sanguineous temperament; acknowledged in many instances the influence of hereditary predisposition; [how this was ascertained, we are at some loss to understand;] or of previous

* See, with reference to the last particular, M. Fournet's experiments with sponge alluded to at p. 303.

† It may not be useless to remark that this observer agrees with MM. Louis, Woillez, &c., in recognizing bulging of the intercostal spaces as a character of emphysematous heteromorphism. Dr. Stokes has the *élite* of observers against him on this point. Vide Br. and For. Med. Rev., Vol. VI., p. 33; and Vol. VII., p. 367.

congestive affections; appeared most frequently in the hottest months of the year, and was in four instances immediately caused by "carbonic acid asphyxia," prolonged muscular effort, or insolation. The local signs were humid viscous respiration (see experiments with sponge); *fall of the inspiratory murmur to* 4 *or* 5, *of the expiratory to* 1°; the ronchus described as humid with continuous bubbles; slight dry cough; slight bronchophonic echo, and dulness on percussion, in extreme cases; sputa rare, white, aerated, slightly viscous; (18 times in 23 cases) all coexisting with a peculiar sensation of discomfort and oppression. Though most commonly developed at the central and posterior part of the lungs, it may occur in any situation, and is neither in its primitive seat nor mode of extension subservient to the laws of gravitation.

Several series of characters, putatively distinguishing this affection from the first stage of pneumonia, capillary bronchitis, passive œdema, and passive congestion, are very industriously laid down; but we cannot help suspecting that M. Fournet's imagination has not been passive in the construction of these well-ordered categories. They are, at all events, such as *à priori* reasoning would easily supply; and *man's* imaginings and *nature's* doings so rarely agree, that we cannot avoid regarding this nice harmony with singular mistrust.

The terminations of active congestion are in resolution, hemorrhage, or pneumonia. The second, it is said, may be either pulmonary or bronchial. The disease merged into pneumonia in eleven of the twenty-three cases, and it is presumable that pneumonic inflammation is always preceded by active congestion: this at least is M. Fournet's opinion; but even analogy does not support it. He conceives the proposition proved, however, by the following result: Having, while intrusted with the care of M. Andral's patients, received two subjects of similar age, constitution, and strength, and both presenting the signs of active pulmonary congestion, he bled one freely, and prescribed some simple ptisan for the other: on the morrow, the signs of congestion had almost completely disappeared in the former, and were converted into those of pneumonia in the latter. This proceeding has been since repeated in a similar manner with a like result. M. Fournet calls it an "innocent experiment;" but, unless this ingenious gentleman has the power of saying to a pneumonia, "Thus far shalt thou go, and no farther," the innocence of the pastime seems somewhat of an assumption.*

M. Fournet's observations on the anatomical characters of active congestion are founded on a single case; they are manifestly in great part those of engorgement, and are completely different from those attributed by Dr. Stokes to the preliminary changes in pneumonia. Dr. Stokes's description has not yet received the sanction of general experience; but that gentleman cannot at least be charged with the absurdity of describing as novel, appearances already familiarly known.

We cannot find room for an account of three chapters occupied with the relation of experiments and hypotheses respecting the connexion of

* M. Bouillaud some time since, as we took care to inform our readers at the period, made the important disclosure that Broussais was the "*Messiah of Medicine.*" Broussais has since departed this life, and, for aught we know, the mantle has fallen on M. Fournet; if so, we must confess it would be heresy to persist in our doubts of the safety of his therapeutical gambols.

the pulmonary murmurs and thoracic movements, the seat and mechanism of those murmurs, and the reason of the coexistence of morbid variations with inspiration or expiration. We confess we regret this but little, as, on the whole, these chapters possess an anticipatory character: they are busy in imagining causes for phenomena of which the very existence is still in many instances problematical.

We shall here take leave of our author's researches on auscultation generally, and introduce the reader to the second part of the work, containing his contributions to the diagnosis of the earliest stage of phthisis. And here, before we enter into a critical examination of these, let us award M. Fournet his due meed of praise for the apparent zeal with which he has entered on his task. If, as Mr. Farr's tables show, 19·6 per cent. of the specified mortality of England is from phthisis, neither the philanthropist nor the medical philosopher can devote their energies better than in searching, by well-ordered observation and close induction, for some method of arresting its ravages. He who shall one day cry "ευρηκα"—and is it Utopian to believe that day will come?—will appear in the records of fame not second even to Jenner.

The incurability of phthisis by art in the second and third periods of the disease is unfortunately a demonstrated fact; but, as M. Fournet enquires, where are the proofs, and who has supplied them, that in its earliest anatomical condition the seal of incurability is stamped upon it? The truth is, they cannot exist; for we have been to the present hour without the means of detecting the presence of tubercles with surety and precision, while yet in their actually incipient state; the opportune time for action slips through our fingers before we are aware of it, and we commence the attack when the enemy is assured of victory. The first point, then, in an enquiry into the possibility of curing tubercle must be an attempt to establish its early diagnosis; and with this view the author has investigated minutely 192 cases of phthisis in the different aspects which the progress of clinical observation and his own special acquaintance with auscultation permitted him to do.

"The existence of the cause is a reason to suspect that of the effect," says M. Fournet; and, building upon the accuracy of this postulate, he examines those agencies which are, for the most part conventionally, fixed on as causes of phthisis, in order to enrol them among the signs of its presence. The justness of this proceeding hinges altogether upon the inseparableness of cause and effect in the evolution of phthisical disease. Now, as with reference to the previous state of knowledge, the idea of such inseparableness is absurd; as the fact of hereditary transmission is almost the only point in its etiology thoroughly established from direct observation on the *human species*, it follows that, unless M. Fournet's enquiries have completely altered the character of that knowledge, his first step in its diagnosis is not remarkable for soundness. This author has 192 cases on which to build his doctrine—192 cases, when thousands have failed to elicit truth! But perchance the defective use of these thousands has been the cause of the failure, and that 192 facts accurately observed and well arranged may succeed where multitudes have only started doubts without settling conviction. Be it so; and let us see how this author has proceeded to avail himself of his limited resources—wherein he exhibits unwonted correctness of plan and execution. He sets out with the principle—of which we grant the truth—that phthisis is either inherited or acquired; and where hereditary transmission cannot be made out, diligently seeks out some anti-hygiological condition to which the patient may have been previously exposed: if successful in his search, he straight concludes that what comes last is the consequence of what went before. Glorious system, this *post hoc propter hoc* mode of argumentation, which tells us, for instance, that night is the consequence of day: so long as we are wise enough to appreciate its excellence, we shall be in no want of etiological instruction. A consumptive patient consults us; we obtain an admission that he has been immoderately given to sexual indulgence, and can discover nothing else to our fancy to account for the disease, therefore that indulgence is its cause; and as it is here a *cause*, in the next individual we meet, whose appetites have similarly sinned, the frailty constitutes a *sign* of phthisis.

But this fundamental vice is not all we have to expose in this chapter; M. Fournet displays marked ignorance of the principles of statistical medicine. He informs us, for example, that a particular form of lymphatic temperament was the most common among his phthisical patients, and hence infers its efficacy in inducing the disease. But this statistician forgets that, in order to justify any such conclusion, lymphatic subjects should not only superabound absolutely, but proportionally to the quota of individuals belonging to each temperament, forming the floating population of the Parisian hospitals in which he observed. If (we of course put the case hypothetically, though the probabilities are in favour of its reality,) lymphatic individuals are more numerous in those hospitals than those of other temperaments, will not the necessary effect be an excess of such patients among the consumptive, unless that form of constitution be actually prophylactic against the disease?

M. Fournet teaches his countrymen that, according to Sir James Clark, the period of life during which the greatest number of phthisical patients are *observed*, is comprised between the twentieth and thirtieth years: now Sir James Clark has not made this assertion, for the table referred to in that pathologist's volume relates altogether to the period of *death*. Though a misquotation, this and numerous other passages show that M. Fournet has not only perused but studied the work in question; yet, with singular hardihood, he produces as original the important opinion that diseases of the digestive and secretory systems of parents are those most prone to influence the health of children. But he inferred the fact from his own researches—possibly he did; but who taught him to search for it? who proved the search would not be in vain? He has no more claim here to the character of a discoverer than the traveller who, provided with the chart of the first explorer of a previously unknown region, follows confortably in the track of his enterprising predecessor.

We have been induced to expose the imperfections of this part of M. Fournet's volume, not on account of any assumed importance of his etiological principles in the diagnosis of phthisis—for he admits generally that little is to be learned in this way, but in order to warn the reader, who in the interest of perusal might forget the fallacious manner in which many of them were obtained, from being imposed upon by the air of scientific exactitude wherewith this author has managed to invest his notions on the causation of the disease. This is the more necessary, as M. Fournet has the *folle vanité* to propose making a book, based on his 192 cases, on the etiology and nature of phthisis. Besides, if, as we conscientiously believe, in minute observation and the numerical method lie the main hopes of improving medicine, it is our first duty to expose such application of numbers to pathology as must tend not only to perpetuate but to give authority to error.

In the author's article on the influence of diseases of the lungs and pleura on pulmonary tuberculization, we find an affirmation that he has several times found tuberculous miliary granulations in the substance of pleuritic false membrane, while the subjacent lung contained not a trace of such product, or simply presented a few granulations or slight tuberculous infiltration of its peripheric substance. He has also seen a large cavern in false membrane similarly situated, while there were only a few traces of commencing excavation in the pulmonary tissue; and known the tuberculous deposition to advance from the false membrane outwards and affect the subcostal cellular membrane only. Under these circumstances the opposite lung, uninvested by false membrane, had occasionally remained perfectly free from tubercle. From these alleged facts the conclusion is drawn, that tuberculization of pleuritic tissue may occur independently of preexisting pulmonary disease; may be primitive and induce consecutively a similar morbid process in the lung. The author's observations tend also, he assures us, to prove that a similar mode of transmission from the serous membranes of the subjacent structure occasionally prevails in the intestines and brain. As we are promised a distinct publication upon this point also, we for the present register it without comment; we shall, on the appearance of the new tome, have occasion to examine the proofs given of the tuberculous nature of the granulations referred to.

M. Fournet's observations lead to the result long since announced by M. Louis, that the existence of *double pleurisy with effusion* involves, in the immense majority of cases, that of tubercles in the lungs. His conclusion on the influence of thoracic inflammations generally on the production and evolution of tubercle is the same as that adopted by Louis, Andral, Dr. Forbes, Sir James Clark, and all those who have observed nature, unbiassed by the dogma of irritation: as to the diagnostic value of intercurrent thoracic inflammations, derived from their peculiar course and character, we perceive nothing novel.

Here is examined at much length the symptomatic bearing of hemoptysis; but the degree of confidence, which the enquiries of preceding observers have taught us to repose in that symptom, is neither increased nor diminished thereby. Its occurrence as the first indication of the disease and in a state of sound health is justly regarded by the author as a circumstance of exceeding rare occurrence. But the fact that in these cases "the aspect of the patient is generally by no means indicative of perfect health," no matter how earnest his own protestations of robustness may be, had already been pointed out to the profession by Sir James Clark.

M. Fournet is of opinion that "active congestion and hemoptysis tend, under the influence of a special predisposition to tuberculous formation, to attract and to concentrate in the lungs the process of tuberculization; and when this is once established to perpetuate and accelerate its progress." These opinions are not new; but as one or two arguments of a novel kind are adduced in their support, we shall put the reader in possession of these. The period of life at which active hyperemia of the lungs most frequently occurs is comprised, as we have seen, between the seventeenth and twenty-fifth years: comparing this result with those obtained in different quarters respecting the age of maximum frequency of hemoptysis and pulmonary tubercle, M. Fournet observes that the period of greatest frequency of hyperemia comes first, then that of hemoptysis, and lastly of tubercle. The catenation in respect of cause and effect is immediately inferred. Now we do not object to the principle by which this conclusion is obtained, but we pray the reader to forget M. Fournet's general terms and attend to the state of the individual facts. He observed altogether twenty-three cases of hyperemia, and yet talks of his *statistical* inferences therefrom : in *two* subjects only did this hyperemia terminate in hemoptysis, in one of these there were already manifest phthisical symptoms, in the other the disease was suspected; and finally, M. Louis has shown that hemoptysis was most common from forty to sixty-five, and in males of nearly the same frequency at all ages. Besides, has it not been proved by M. Louis, that the existence of tubercle in any organ in subjects past the age of fifteen, involves almost with mathematical certainty their presence in the lungs; in other words, that those organs are essentially the natural primary seat of the morbid formation, and this without the influence of any previous hemoptysis to "attract the process of tuberculization to them?"* But while we deny that these clumsy attempts at numerical induction prove in the least the point on which they are intended to bear, we do not intend to question the probable secondary influence of congestive movement on tuberculization.

The possible production of heterologous substances generally in the interior of fibrinous coagula is a question of high import. M. Andral, on the evidence of a single case, originally maintained the possibility of the direct transformation of an apoplectic clot in the lung into tuberculous matter. Criticised by M. Meriadec Laennec, he acknowledged his inference had been premature; and has never since met with a similar appearance in the lungs, but still affirms that such is the apparent mode of origin of splenic tubercles. Our author considers M. Andral's concession uncalled for, because he has himself observed a fibrinous mass in the centre of a clot in the aorta, studded with and almost wholly formed of small whitish, nonvascularized granulations. It is not a little curious how important a part the fibrinous portion of clots has been made to play by different writers: M. Andral, finding what he took for pus thus situated, in an instant converted the containing coagulum into an absorbing and secreting agent, endowed it with life, and with this *fact* for his text,

* M. Fournet marvels elsewhere why, in a subject addicted to sexual debauchery, the lungs, which had not been locally excited, were the principal seat of the disease; the testes and penis no doubt *ought* to have been the chief sufferers. But he most philosophically masters the difficulty by hinting, that the acceleration of respiration accompanying the act of coition may have been the cause of the pulmonary deposition!

sermonized on the wonders of organization: the microscope has happily dispelled those crude imaginings by showing the presumed pus to be nothing more than some of the substance of the clot itself in a softened state. What ingenuity, again, has been wasted in explaining the origin of the central "pus" of the coagula of phlebitis. And now we are required to credit a doctrine of tuberculization founded, the probabilities are a thousand to one, on some similar illusion. We know that an anatomist of somewhat greater experience than our author, namely M. Cruveilhier, was deceived into the belief that he had produced tubercles by injecting mercury into the bronchi; he mistook pus for that morbid matter. When our advocate of fibrinous tuberculization provides himself with some half dozen cases in which the *microscopical* character of the alleged tubercles are accurately enumerated, we shall give him a patient hearing.

Before entering into M. Fournet's exposition of the physical signs of the first stage of phthisis—a subject on which he is entitled to a greater share of attention than on those just adverted to—it is natural to enquire how these were ascertained; as the affection does not usually cut off its victims at so early a period of its existence as to allow a comparison of the signs of that stage with its anatomical characters. First, this observer carefully noted the signs produced on the confines of manifestly diseased parts, and compared these signs with the incipient disorganization by which they had been induced. Secondly, he traced the gradual advancement of the disease to the rest of the lung in patients presenting the signs of the second stage in the upper portion. Thirdly, he followed the gradual changes undergone by the respiratory murmurs in patients admitted into the hospital for affections unconnected with the chest, but who therein became phthisical, and presented a successive series of signs and symptoms, from the most trifling and obscure to those of excavation. Such being the judicious mode (for the possible fallacies likely to spring from one method are avoided and rectified by the others) in which the signs are stated to have been ascertained, we proceed to lay an abridged account of these before our readers.

*Respiration.* The *inspiratory* murmur increases in intensity to 12, 15, or 18; though less uniformly, diminishes in duration to 8, 7, 6, 5, and becomes dry, hard, and rough. The latter character is invariable in its existence, until marked by alterations of *quality;* the former two are not unfrequently absent. The *expiratory* sound undergoes an increase in intensity and duration by successive steps from 2 to 20; both characters in the majority of cases alter *pari passu*, but in rare instances the intensity remains unaltered, though the prolongation is marked: a sensation of dryness and hardness is at the same time conveyed to the ear. The extent of alteration of both murmurs furnishes a tolerably exact estimate of the degree of advancement of the first stage. These changes are accompanied with modifications of *quality*, which, exhibiting themselves first in the expiratory murmur, successively pass through the ascending series already described, the first four grades corresponding to the incipient stage of the disease. While the change of *quality* belongs to one or other of these four grades, it invariably presents itself in a more intense form in expiration than in inspiration; the cavernous and amphoric qualities, on the contrary, affect both murmurs to nearly a similar amount. With respect to the order of development and respective importance of these changes, it may be stated that modifications of duration and intensity present themselves first, and are therefore the very earliest physical results of tuberculization. Modified duration and intensity of the expiratory murmur, and changes of *quality* in both, have respectively much greater diagnostic value than similar alterations of the inspiratory sound, or than a dry and hard character in either murmur. The morbid variations now passed in review generally coexist with each other; so true is this that were some of them detected without the others, their dependence on tubercles should be called in question. It is important to add that to the description now given M. Fournet has encountered but very rare exceptions.

*Ronchi.* The ronchi of the first stage of phthisis are stated to be *the pulmonary crumpling sound, and the dry and humid crackling.* The former we have already minutely described; we shall now similarly dispose of the latter. The two crackling ronchi are successive degrees of the same phenomenon, the first accompanied with a character of dryness, the latter of humidity. The dry species is composed of a series of two or three crackling sounds; the humid form is similarly constituted, but its component parts gradually acquire the bubbling character more and more distinctly, until they actually produce a bubbling ronchus. The drier it is, the more completely is it confined to the inspiratory movement, when humid it accompanies both murmurs. The seat of this ronchus is primitively the summit of the lung, but it always originates, wherever there is tubercle in the first stage. The dry form is generally heard over a less extensive surface than the humid. Once developed the crackling ronchus rarely disappears temporarily (it did so in 9 out of 55 cases), but is distinguished by a persistence, which is itself more marked the longer the ronchus has existed. While possessing the dry character it is peculiar to tuberculization, and can be confounded with no other sound even in respect of its audible properties: the humid form may be mistaken for mucous ronchus, if its acoustic characters only be taken into account, but the course and seat of the phenomenon will clear up any doubts as to its nature. The crackling ronchus does not appear until the respiratory murmurs have undergone some slight alteration in intensity and duration, and, while other changes of *quality*, &c., seize those murmurs, passes successively to the humid form, the bubbling, the cavernulous, and the cavernous or gurgling. The time occupied in this series of mutations varies: M. Fournet found, in the majority of cases of acute phthisis, that the passage from the dry to the humid form was effected in from eight to twenty days; in chronic tuberculization in from twenty days to three months. The dry form occurs when a good number of crude tubercles either isolated or collected in groups form the anatomical evidence of the disease; under peculiar circumstances, however, it would appear that a very few minute tubercles scattered through the pulmonary tissue are capable of giving rise to it.

The pulmonary crumpling sound is far from possessing the important diagnostic bearing of the ronchi we have been examining; and really signifies nothing, unless coexistent with modifications of the respiratory murmurs; but if thus detected, it augments the surety of the diagnosis precisely at the period, the first or second phases of the first stage, when the difficulty of detecting the disease by its sensible signs is greatest. The change to the humid character announces the establishment of softening, and the advancement of this process is measured by the degree of humidity, and rapid or slow assumption of the bubbling character. Sonorous and sibilant rônchi depending upon local bronchitis sometimes coexist in the same region with the phenomena just described.

The regularity of occurrence of the changes traced by M. Fournet is of course a most important element for consideration in their diagnostic utility. He appears to regard those appearing to affect the inspiratory murmur as inseparable from the presence of tubercles, and gives it as his belief that if the dry crackling ronchus be not always observed, this arises from an examination not having been instituted at a sufficiently early period. We have already mentioned his ideas regarding the immediate seat and mechanism of the crackling ronchi; by others the humid form, at least, has been supposed to originate in the minute bronchial tubes, and such seems from the following passage to be the decided opinion of Dr. Hughes: "There is not unfrequently added to the signs already mentioned, either a small thin mucous rattle, resulting from the admixture of air with the glairy fluid existing in the minute tubes, or a single clicking sound, arising apparently from portions of thick viscid mucus, presenting an obstacle to the passage of air through those of larger caliber."* We may here notice that Dr. Hughes speaks of "the bronchial irritation preceding the formation of tubercles," as if such were an usual or even a constant phenomenon. He appears to be ignorant that M. Louis long since proved that the bronchi are as frequently healthy in phthisis, except when communicating with *softened* tubercle, as in individuals dying of all diseases indiscriminately; and that Major Tulloch has shown by the irrefragable testimony of figures, that while 13 per thousand of the whole military force are annually under treatment for consumption in *Jamaica,* and only 5 or 6 per thousand *at home*, catarrhal complaints only affect 65 per thousand of the force in the former place, while they are 122 per thousand in the latter. Pneumonia is still more rare in Jamaica.†—But to return to the signs of phthisis.

*Voice.* With the second phasis of the first period, in about one half the cases, a peculiar change occurs, according to M. Fournet, in the characters of the voice, rendering its tone grave, its quality less clear, its general character veiled, pectoral, and slightly hoarse: the whole constituting the incipient state of the well-known peculiar condition of the voice of subjects in an advanced stage of the disease. This change appeared to advance with a degree of rapidity proportionate to the frequency and duration of the colds under which the patient had suffered.

We observe nothing of the least novelty in this observer's analysis of the auscultatory phenomena. M. Fournet gives the recommendation to make the patient, instead of answering desultory questions, count a cer-

* Guy's Hospital Reports, No. ix.

† Vide British and Foreign Medical Review. Vol. VIII. p. 220.

tain number of figures, while the comparison is instituted between the resonance on both sides. In this way the changes of intonation produced by different words, and which probably affect the phenomena in question in a slight degree, are avoided. We have long been in the habit of employing this plan, (proposed, if not by Laennec, in his time,) but not, we confess, with M. Fournet's motive, which seems to us important.

On *cough* and *the transmission of the heart's sounds*, as indicative of tuberculization, there is nothing to detain us: the transmission of subclavian murmur by solidified lung, first noticed by Dr. Stokes, has escaped M. Fournet. There seems, however, to be some doubt as to the exact period of the disease at which this phenomenon becomes perceptible. By *percussion* no appreciable change of sonorousness is detected until the second phasis; on the application of this method of diagnosis, the volume before us falls short of the productions of Piorry, Dr. Stokes, and others.

Under the title *acouophonia* or *cophonia*, M. Donné has designated a mode of investigation in which the observer places his ear to the chest and analyzes the sounds produced by percussing different parts of that cavity. This plan would perhaps appear better fitted *à priori* than any other to furnish a correct notion of the different degrees of density of the contained organs. But as M. Fournet has found by experience that it is really deceptive, that the perceived sound bears no precise relation to the condensation or rarefaction of the subjacent parts, we shall not seek to rescue M. Donné's novelty from the oblivion into which it is fast subsiding.

The modifications of locomotive and vibratile movement of the chest ascertainable by *palpation* or manual examination are delicately analyzed by the author; though the result, as respects the former, is rather to be noted from its declared connexion with the first period of phthisis and diminished volume of the lung, than from its newness. "In cases of tuberculization inducing a notable increase of density in the summits of the lungs, the *partial motion* of the corresponding ribs undergoes a diminution proportional to the induration and decreased size of the organ. The *motion of totality* (of general ascent and descent of the thorax) is, if at all modified, rather augmented than lessened in extent. As this sign cannot, generally speaking, be detected, until the disease is sufficiently advanced to be readily discoverable by auscultation and percussion, it might be considered a diagnostic superfluity, did it not seem to possess more negative than positive value. As in cases of disseminated miliary tuberculization the costal movements are not sensibly affected, the absence of morbid change in those movements may become a positive sign of that particular anatomical form of phthisis, when the other physical signs and the symptoms announce an advanced period of the first stage.

To us it has always appeared exceedingly difficult to draw any satisfactory conclusion from what is termed the vibratile motion or fremitus of the thoracic parietes. We shall probably be told that our tactile perception is of imperfect quality; and we should submit to the aspersion without demur, did we not detect a very notable contradiction on the subject in the statements of observers who, we presume, deem their fingers most happily moulded. If we believe Dr. Williams, liquid in the pleura will abolish the natural vocal fremitus completely, while in cases of hepatization, and under all circumstances of consolidation, the vibration is "*much stronger than on the healthy side.*" According to Dr. Stokes, the *absence* of vibration over the dull portion of surface is an exceedingly useful sign in the diagnosis of pleural effusion, *hepatization*, and enlarged liver, though traces of vibration may be detected in some cases of hepatization. But a very different version is that of M. Fournet. This observer affirms in the most satisfied tone that the maximum of vocal and tussive fremitus obtains in the normal state of the organ; that the phenomenon is always in the inverse ratio of the *density* or *rarefaction* of the pulmonary tissue and is totally abolished alike in hepatization and advanced emphysema. As these latest observations appear to have been made with patience, the following *excerpta* from the general conclusions respecting the healthy fremitus may possess interest for some of our readers. The vocal vibration is stronger in decumbiture than in the erect position; when the individual observed speaks in monosyllables than in polysyllables; in the front than in the back of the chest, on the right than on the left side anteriorly (noticed by Dr. Stokes also), most strongly developed in the lower part of the sternal region; extremely little marked in the clavicular region, hardly better in the sub-clavicular zones; and in many subjects altogether wanting. The difficulty of appreciating slight modifications in a sign thus characterized is sufficiently obvious; but M. Fournet's enthusiasm is nothing daunted, for he affirms that if the hand be laid over a mass of lung partly healthy, partly diseased, "the difference of vibration corresponding to the different parts of the hand may be *easily* distinguished." With such perfect specimens of tactile organization as this gentleman, it is of course vain to argue. It follows, however, from the general position above stated, that, as indeed is elsewhere distinctly affirmed, the vibration is diminished opposite tuberculated lung in the exact ratio of the extent of disease: an idea opposite as night to day to that promulgated by Dr. Williams. We are further told that a very slight difference in density of the summits will induce a manifest difference of vibration; yet this situation is pointed out as one distinguished by the very trifling degree of vibration producible in it when at the maximum, namely, in the normal state of the lungs. Why, if M. Fournet's statements be correct, the laws regulating the decrease of *tactile* should differ so widely from those of *acoustic* vibration we are unable to comprehend.

The author's enquiry into the signs of the first stage of phthisis, furnished by *inspection*, opens with an enumeration of some of the principal facts thus ascertainable in healthy subjects. The only point here stated bearing on this particular subject, which had not been elicited by the researches of M. Woillez,* seems to be that a piece of tape, stretched

* M. Fournet, with his usual happy ignorance of the proceedings of his contemporaries, knew nothing of these researches, of which an account was first published in May, 1835, until "he set about publishing his own work" in 1839! Truly the flood of light, which burst on M. Fournet from all quarters at that particular moment, must have been dazzling. Here, too, he manages to show us how easy it is to descry the from the nipple to the most prominent part of the corresponding clavicle, will lie in immediate contact with the skin—a fact of which we shall presently learn the application.

The uniform transverse retraction of the upper portion of the chest, which induces the well-known cylindrical form of thorax, may, according to M. Fournet, be observed before or after the deposition of tubercle. This we admit; general experience acknowledges the fact, and M. Woillez has proved it. But we question if such preexisting conformation indicates, as the author affirms that it does, a predisposition to the growth of tubercle; the accurate observer just named has, it is true, shown that such malformation is much more frequent in phthisical subjects than in others, but where is the proof that in the particular individuals furnishing *this* result, the thoracic malformation preceded the tubercles in order of development? M. Woillez, we are aware, classes the defect of form in question with his physiological heteromorphisms; but as he appears to have been ignorant of the atrophy, occurring in tuberculous lungs, which distinctly leads to sinking in of the parietes, we cannot accept his decision in the matter. This species of contraction exists, according to our author, in one fourth, or at the utmost one third, of phthisical subjects; he estimates as follows the diagnostic bearing of different degrees of this deformity. If we discover such contraction in a subject presenting the other signs of the first phasis of the first stage, it must evidently belong to the preexisting species. In the second phasis, if the deformity be much developed, the inference will be the same; but in the third, we may fairly conclude that such abnormal appearance is an *effect* of tuberculization. The preexisting may, it is said, usually be distinguished with ease from the consecutive contraction, in the following way: the former species exists to the same amount on both sides of the chest, and in its entire height, though gradually diminishing from above downwards; the latter is particularly observed on the most diseased side, and at the summit. All this looks amazingly clear on paper, but we strongly doubt that these distinctions are founded on observation, or that the author has ever come to a conclusion in practice from their application. In justice we must add, that he does not regard this heteromorphism as possessing absolute diagnostic force, but merely as warranting suspicions, which it is the business of the other signs to confirm or invalidate: to maintain a different opinion would be absurd, for this precise conformation is observed in persons who descend to the grave without a tubercle in their frame.

M. Fournet again proclaims his literary deficiency, by affirming that mote in our brother's eye, while we dream not of the beam that obstructs our own. He objects to M. Woillez's conclusion, that individuals following trades, which require particular exercise of the upper extremities, have on an average less development of thorax than others, because it is founded on too limited data (133 cases). Yet he himself preaches the law on every possible point of the subject of phthisis on the foundation of scarcely a greater number of observations, resolves with the most perfect composure problems which the wisest statisticians regard as insoluble in the existing state of national censuses, and deduces *statistical* inferences from *twenty-three facts*. But the true cause of his objection is, that the conclusion in question opposes some of his favorite theories.

depression of the sub-clavicular region has not been noticed in cases of phthisis, except as a consequence of pulmonary excavation, and in supposing himself original in the announcement that it may be induced by crude tuberculization. "Decrease of the antero-posterior diameter of the thorax, with flattening or slight hollowing under the clavicles," says Dr. Stokes, "may be appreciated at a much earlier period than has been supposed..........and is found a most important aid in diagnosis *in the earlier stages of phthisis.*" But this modification has been more minutely studied by M. Fournet than his predecessor. In every subject examined after death in whom he had observed it, the summit of the lung was found to be invested by a fibro-cellular false membrane uniting it closely to the adjacent wall of the chest. The sign is not, as he believes, available until the close of the first period, but he has occasionally observed it at the outset of the disease, when preexisting pleurisy had led to the deposition of firmer and more resisting pseudo-membranous tissue at the summit of the lung than elsewhere. This sunken appearance may usually be detected by simple inspection, but we are recommended to measure its degrees, by stretching a piece of tape from the nipple to the clavicle : the distance intervening between it and the thoracic surface gives the measure of the deformity. Dr. Stokes uses a pair of callipers moving on a graduated arc, one branch of which is fixed on the scapula, the other below the clavicle; this method will also give the measurement of any posterior depression which may be present.

M. Fournet adds nothing to Sir James Clark's accurate description of the visible changes in the expansion of the ribs opposite tuberculous accumulations ; he states, however, that he has not observed this species of change except when there was sub-clavicular depression, and that both these signs may be detected in about one fourth of phthisical subjects.

Having accomplished his task in respect of the physical signs, M. Fournet turns to the local symptoms of the first stage. Admitting the observation made by Kuhn, that the microscopical *tuberous tissue*, of which tubercle is, according to that writer, composed, may be detected in the sputa of the consumptive, he affirms that the fact cannot be available for the purposes of diagnosis, until the period of commencing softening; as before that change, the passage of tuberculous matter into the expectoration cannot be supposed to take place. But this is an assumption which, though probably well founded, requires the attestation of direct experience; it is irreconcileable with Dr. Carswell's opinions respecting the seat of tuberculous secretion.

In speaking of dyspnœa as a symptom of the incipient stage, M. Fournet avails himself of M. Louis's cases, as countenancing the opinion that that symptom, when originating in early life, proceeds from the fancied imperfection of pulmonary development already spoken of. But he omits to mention, that M. Louis himself has ascribed it to the coexistence of emphysema with the tubercular disease.* The description of this symptom generally adds nothing to the graphic sketch of Sir James

* British and Foreign Medical Review. Vol. VI. p. 38.

Clark; the notice of the dyspnœa of acute phthisis may, however, be usefully consulted.

Regarding the seat of tuberculous accumulations in the lungs, M. Fournet touches upon a point respecting which a curious difference obtains in the statements of observers—we mean the relative frequency with which the two lungs suffer and present the greater share of disorganization. As this is not a point of mere curiosity, but one which has close connexion with the application of the principle of comparison in diagnosis, it deserves investigation. The majority of writers assert that the left organ is most frequently and extensively implicated; Laennec seems to have stood almost alone in maintaining a contrary opinion. M. Louis, among others, has given the authority of his recognition to the former notion, and is cited to this effect by M. Fournet and others; but, curiously enough, the cases of that observer do not, on analysis, warrant his inference, for here is the result in figures of his fifty tuberculous subjects:

| | | | |
|---|---|---|---|
| Cavities in both lungs in 33 cases | Most extensive in right lung in | 13 | cases |
| | left | 12 | — |
| | Doubtful in this respect | 8 | — |
| Cavity in left lung only | | 7 | — |
| right | | 6 | — |
| Tubercles (without excavation) predominating on the | right side | 1 | — |
| | left | 1 | — |
| | doubtful on which | 2 | — |
| | | 50 | |

Now both lungs appear to have suffered as closely as possible to the same amount. Of 170 of M. Fournet's patients, 109 presented the greatest share of disease on the right side, forty-six on the left; in fifteen cases both organs were equally affected. M. Hirtz also speaks, in his recent thesis, as if he had obtained a similar result. As a small minority, twenty-nine only, of our author's patients were examined after death (although the ratio was among these as 21 : 8 in favour of predominance of the right lung), the question must be allowed to stand in need of further examination. Whatever be the decision, we, however, see no objection to conceding that it is unusual to find the disease equally developed on both sides—indeed, the tabular view we have given of M. Louis's cases proves this. The author elicits no novelty in his considerations founded on this fact; Dr. Stokes appears to have exhausted the topic.

Before extracting some passages from the author's estimate of the value of the physical signs, we shall exhibit, tabularly, the physical signs of the three phases of the first stage; we shall thus, we conceive, facilitate the reader's comprehension of the subject generally. In drawing up this synopsis, we have noted each phenomenon with the characters and in the position most frequently assigned to it by M. Fournet.

| | | |
|---|---|---|
| *First Stage.* | FIRST PHASIS.<br><br>A few small tubercles scattered through the lung. | Inspiration, dry, rough.<br>intensity increases to 12.<br>duration falls to 9, 8.<br>quality, natural.<br>Expiration, dry, rough.<br>intensity, } duration, } rise gradually to 8.<br>quality, clear, ringing.<br>Commencing bronchophony in rare cases. |
| | SECOND PHASIS.<br><br>Infiltration of crude tubercles in groups. | Pulmonary crumpling sound.<br>Dry crackling ronchus.<br>Sonorous, sibilant, ronchi (symptomatic of bronchitis).<br>Inspiration—intensity = 12, 14.<br>duration = 9, 8.<br>quality, clear, ringing.<br>Expiration, intensity } duration } = 6, 10.<br>quality, blowing, rarely bronchial.<br>Dryness and roughness of respiratory murmurs are now masked by change of quality.<br>Slight bronchophony, frequently.<br>Slight obscurity of sound on percussion.<br>Diminished vocal fremitus.<br>Unnaturally distinct transmission of cardiac sounds. |
| | THIRD PHASIS (or of transition from first to second stage).<br><br>Commencing softening. | Humid crackling ronchus.<br>Sonorous sibilant ronchi, as before.<br>Pulmonary crumpling sound disappears.<br>Inspiration—intensity = 15, 18.<br>duration = 7, 6, 5.<br>quality, blowing, or slightly bronchial.<br>Expiration, intensity } duration } = 12, 15, 18, 20.<br>quality, bronchial.<br>Strong bronchophony, or imperfect pectoriloquy.<br>Sound more obscure, or even dull.<br>Vocal and tussive fremitus much diminished.<br>Diminution of partial movements of ribs corresponding to indurated mass.<br>Transverse retraction of corresponding part of the chest.<br>Subclavicular flattening. |

But the facility with which these signs may be ascertained, the constancy of their presence, the regularity of their progress and catenation, form almost as important elements in judging of the confidence to which they are in practice entitled, as the actual reality of their existence. Upon some of these points, in so far as they are capable of being generalized on, M. Fournet speaks with just appreciation of the difficulties of clinical medicine.

"The observer must, while investigating the local signs, carefully guard against being influenced by any preconceived opinions, originating in the external appearance, or in the commemorative or general symptoms of his patient. If dominated by an opinion, in great measure already formed, the very best auscultator is not unlikely to become so careless and inattentive in his examination, that the impressions received by the senses are, without his being aware of it, converted into so many confirmations of his preformed judgment. This is the more likely to happen, because the phenomena appreciated by the senses and the mind are very numerous, and only distinguishable by very delicate differences from others of wholly distinct diagnostic force. When a first examination has been made, under circumstances like these, we are sometimes astonished at the variation in the result of a second. . . .

"The physical signs are not, as might be believed, pathognomonic of phthisis; for such signs alone merit that title as belong exclusively to a certain anatomical condition. . . . Some observers consider a prolonged expiratory murmur peculiar to phthisis, and almost all that has been written on the sound of expiration bears the impress of this idea; the belief is a groundless one, for the expiratory murmur manifests itself in a multitude of different conditions. . . . But from mutual combination, from coexistence with some signs, and from their appearance, independently of others, the phenomena described may lead to a precise diagnosis of tuberculization; their primitive or absolute value goes no further than pointing out the presence of induration or foreign bodies in the pulmonary tissue. . . .

"At a very early age no inference could, I think, be drawn from the existence of the morbid characters of the respiratory murmur, which I have assigned to the first phasis,—in many instances even to those of the second." (p. 263, et seq.)

Fully as we assent to the justness of these remarks generally, we would, nevertheless, point out a slight contradiction between one of them and the author's previous assertion, that the dry crackling ronchus is peculiar to tuberculization,—if peculiar to, it must, when present, be pathognomonic of the disease.

Nothing can be clearer than that the likelihood of forming a correct diagnosis through the physical signs increases, the more advanced the phasis of the first stage during which the examination is made. We shall not be so traitorous to the principles we have elsewhere professed as to affirm that the diminution of confidence in them, during the first phasis, must often amount to negation, unless they receive strong confirmation from the general and commemorative symptoms; but we should ourselves be disinclined to trust them, unless so supported. We, in truth, in this opinion echo, as we shall presently see, M. Fournet himself. Who shall affirm his capability of surely estimating changes of intensity and duration, amounting only to one or two tenths of the normal sum—who, if persuaded that such delicate variations are estimable by his senses, can declare, that such departure from the normal mean may not be the effect of individual idiosyncracy? And again, is it easily credible that the complicated system of respiratory changes and ronchi, exhibited in the above column, works with the precision and regularity of a mechanical contrivance—that a morbid change once set in action never vacillates, but continues its onward course without interruption, till it reaches its maximum? Assuredly it is not; and however M. Fournet's general tone may be calculated to inspire conviction of the regularity of these changes, he is honest enough to confess, occasionally, that exceptions occur. In fact, the division into precise phases, and the ascending and descending notation of respiratory duration and intensity, are to be taken as approximations to the truth; unless this be admitted, numerous trifling contradictions, which escape the author in different parts of his volume, cannot be accounted for. But whatever be the irregularity to which it is probable the course and progress of these signs are liable, the reader must not forget that there scarcely exists a known combination of symptoms in any disease secure from similar unsteadiness.

Our author has, he alleges, observed, that the pulse of a great number of phthisical patients presents, even during the first stage, a peculiar softness. To this observation he, however, attaches little importance; and we notice the point merely to introduce to the reader a curious disclosure, recently made by Dr. Guy, respecting the diagnostic information to be derived from the pulse at an early period of the disease.* We have space for the conclusions only, but the striking character of these will, we doubt not, attract our readers to the original paper.

"1. In cases of phthisis pulmonalis the frequency of the pulse varies within wide limits; the difference between the extremes amounting to 90 beats.

"2. In the same individual, the frequency of the pulse undergoes remarkable fluctuations, passing, in a few days, through a range of upwards of 60 beats.

"3. In five out of six cases, the frequency of the pulse in phthisis exceeds the highest frequency observed in health.

"4. The difference between standing and sitting in phthisis is nearly the same for all frequencies of the pulse.

"5. The maximum difference between standing and sitting in all cases of phthisis pulmonalis, falls short of the mean difference in health.

"6. From the average results of a considerable number of cases, it appears that the mean difference in health is six times as great as the mean, and three times as great as the maximum difference in phthisis.

"7. *On the supposition that the slight effect produced by change of posture is peculiar to phthisis pulmonalis, it forms one of the most constant and certain of its symptoms.*

"8. *On the supposition that the slight effect produced by change of posture* is common to more than one disease characterized by increased frequency of pulse, it will distinguish these diseases from others with which they may be confounded; and these diseases themselves are easily distinguished from phthisis pulmonalis, either by the peculiar character of the symptoms or by other physical signs."

We fear Dr. Guy forms too favorable an estimate of the diagnostic utility of the "slight effect produced on the pulse by change of posture in phthisis;" should experience prove us wrong, he will have fairly distanced M. Fournet, for, certainly, there are more persons capable of counting to 100 than of appreciating the delicate phenomena described by the latter observer. It is obvious that Dr. Guy is only entitled to affirm, from observations hitherto made, that the influence of posture is different in health and in phthisis; he cannot at present declare, that in many other diseases the pulse is not similarly affected, as in consumption. In his own practice he evidently sets at nought this difficulty; for he gives an instance of his diagnosticating phthisis on the evidence of this condition of pulse, and such appears, from the narration, to be not very uncommonly his habit. In the seventh and eighth propositions, a sort of provision is made for the objections of those unwilling to go the length of Dr. Guy, upon the imperfect data recorded.

But to return to M. Fournet. This observer next presents us with a close investigation of the course and progress of the disease. The question, whether the local or general symptoms appear first, is one of no mean importance; it is one, also, on which we are willing to hear M. Fournet, because he appears to have comprehended fully, and, as far as possible, eschewed the circumstances likely to involve erroneous inferences on the subject—we mean imperfect analysis of the local signs of the earliest phases of the disease, and careless interrogation of patients

* Observations on the Pulse in Phthisis Pulmonalis. By Dr. Guy, Guy's Hospital Reports, Oct. 1839.

regarding the symptoms they may have experienced at or previous to the supposed outset of the complaint. His conclusions are, that in essentially *chronic* phthisis, the sensible phenomena of tuberculization may be sometimes detected in individuals presenting, in a marked manner, the evidences of predisposition to phthisis, at a period when neither they themselves, nor the medical observer, can discover any general symptoms; that in other cases, the constitutional symptoms lead the van, at a period when, though demonstrative proof cannot be given that microscopical tuberculous matter does not inhabit the lungs, it is yet, if it really does exist, in too minute quantity to affect, in anywise, the respiratory murmurs; that, in a third series of subjects, the local and general manifestations appear to originate simultaneously. In *acute* phthisis the general phenomena appear to set in at least as early as the local changes. These general statements may be considered to leave the matter just as their author found it, and, for the reason we have already given, some confidence may be placed in them. In commenting on the second variety, M. Fournet observes, that the general phenomena cannot be considered a mere effect of the reaction of deposited tubercle on the system generally, but that, in a number of cases, "they appear to be the direct effect of the tuberculous cachexia." This is evidently appropriated, without acknowledgment, from Sir James Clark, and, at the same time, unskilfully borrowed, for under these circumstances they are not the effect, but the actual essence of the *cachexia* itself, as it is termed by our distinguished countryman.

We need not dwell upon the author's description of the course of the symptoms and signs; the order of development of the latter is distinctly shown in the table we have given of them; the account of the former is a periphrasis of Sir James Clark's chapter on the subject, and, indeed, corresponds therewith to a marvellous degree in some of its phraseology.

Two chapters and forty-nine pages are given by the author to the *circumstantial* and *differential* diagnosis of the affection. Under the former of these heads, he presents us with various combinations of local and general symptoms, which may occur in individual cases, and endeavours to estimate the probabilities for and against the existence of phthisis furnished by each. Here is exhibited much clinical sagacity, as, we think, the following specimens, selected chiefly from their reference to the value of the physical signs, will fully show. It is necessary to premise, that the accuracy of the diagnosis, under the circumstances supposed, has, in almost every instance, been ascertained, either by post-mortem examination, or by the gradual passage of the disease to its most advanced stages.

"CASE I. Being given an adult patient of strong constitution, sanguineous temperament, originally of robust health, of healthy parentage, never having been subject to colds or cough, nor having had hemoptysis, but who, *after exposure for a certain time to anti-hygienic influences*, perceived a change in his state of health, we may, with perfect confidence, diagnosticate phthisis, provided we can detect distinctly and persistently the sum of local and general signs attributed to the second and third phases of the first period, and if there exist no other morbid condition capable of accounting for these signs.

"CASE II. Under the same circumstances, the same diagnosis may be made, but with less certainty of correctness, if the local signs be only those of the first phasis; provided the whole series of signs and symptoms acknowledge precisely, or nearly so, the laws I have traced as ordinarily regulating the development, course, and symptoms of accidental or acquired phthisis.

"CASE III. If, under the circumstances supposed, the general symptoms were wanting, the local signs of the first phasis would only warrant a simple *suspicion* of the existence of phthisis. Again, if the general symptoms were absent or of doubtful character, and the signs of the second phasis existed in a distinct and regular manner, the fact of tuberculization would become *probable*. Finally, if the local signs of the third phasis are observed in a marked degree, and although there be no distinct general signs actually present, but something suspicious in the character of those, either existing or not long past, we may be *pretty certain* of the presence of pulmonary tubercles. . . . .

"CASE X. The existence of the general symptoms of hectic fever, which I have said belong to the first, second, or third phases of the first period, if unaccompanied with any of the local signs of that period, could not, even if there were hereditary predisposition to the disease, justify us in concluding, that phthisis was actually present; but would warrant the opinion, that tuberculization will sooner or later take place. If, in addition to labouring under hereditary predisposition, the patient have had hemoptysis, colds, and cough, with the course observed in phthisis, the absence of local signs should be set down as an exceptional occurrence, depending on a particular organic condition of the lung, and the case considered one of phthisis. . . . .

"CASE XIV. If a subject born of apparently healthy parents, of only moderately strong constitution, and possessing delicate health, who has had frequent colds and chronic cough, and presents the morbid state of the voice elsewhere described, were attacked with pleurisy with effusion, which effusion becomes chronic, without any apparent cause for such termination, and entails deterioration of the general health, that subject should be considered affected with tubercles."

A similar position had already been established by M. Louis; but its vast practical importance justifies its repetition, whenever an opportunity occurs.

"CASE XIX. When the ordinary signs of the third phasis of the first period, ascertained by *auscultation and percussion*, exist only *on one side*, while natural respiration and sonorousness are detected on the other, the idea that the case is one of phthisis should be rejected, unless the signs furnished by *inspection* were exceedingly manifest on the diseased side. Experience shows in truth that, unless in the instance of a thick false membrane investing the summit of the lung, tuberculization, of the amount necessary to produce the physical signs supposed, *does not exist on one side only*." (p. 761, et seq.)

With the extensive chapter, next introduced, on the prophylaxis of the disease, we shall not delay; for, though its contents exhibit favorably the author's capabilities for the discussion of such questions, and are remarkable for their excellent arrangement and lucid combination, those acquainted with Sir James Clark's volume (from which, indeed, they seem largely borrowed,) can here discover no new principle started or developed. In the details, some novel notions occasionally break forth; but the youth and necessary inexperience of M. Fournet in the treatment of disease, deprives these of the sterling quality for which the English practitioner would value them.

Our best authorities, M. Andral, Sir James Clark, &c., hesitate in their judgment respecting the possibility of contagious transmission of phthisis; the following account, bearing on this question, is sufficiently curious to merit extraction. Its accuracy rests on the authority of M. J. Guérin, the orthopædist.

"A female died in the third stage of phthisis, having shared her husband's bed till her last moments. The latter, who was originally of robust constitution, and sprung from a family, none of the members of which had ever been phthisical, married a second time; the subject of this union was of good constitution and healthy parentage. Eighteen months after marriage he died of confirmed phthisis; his wife, who cohabited with him to the last, remarried shortly after, and in two years died of consumption. Her second husband, of strong constitution, and belonging to a family in which phthisis had never been heard of, perished of the same affection soon after the death of his wife."

Upon this tale we shall only remark, *se non è vero, è ben trovato*, for the contagionists.

A disquisition on the curability of phthisis, during its third and first periods, presents some novel and speciously supported opinions. M. Fournet admits the possibility of a natural cure during the third stage, but endeavours to show that Laennec was in error, both in respect of the frequency of its occurrence and in ascribing the fact to the cicatrization of caverns. Curiously enough, this doctrine is put forward at the precise period that another Parisian observer, M. Rogée, publishes an elaborate and needlessly prolix paper, to prove that pathologists have no conception of the vast number of cases thus cured. The substance of M. Rogée's paper is elsewhere given; and we must here limit ourselves to an abstract of M. Fournet's arguments: to institute a critical comparison between the rival doctrines would occupy more space than we can devote to the subject. Our author commences with a verbatim exposition of Laennec's theory of cicatrization, from the works of that observer, and then describes the very different manner in which he views the same phenomena. He remarks, founding his statements upon the dissection of lungs in which no distinct trace of tuberculous disease was discoverable, that when pleurisy with effusion takes place, leading to compression of the lung and exudation of membranous matter, if the false membrane is thicker and firmer in some parts than others, the surface of the lung is always depressed and furrowed in the corresponding spots. The furrows thus produced are sometimes so much as an inch in depth, and always proportional in dimensions to the general or local decrease in size of the lung. The false membrane on the pulmonary surface sends prolongations of different extent into the interior of the sulci. At a late period, the superficial patches of pseudo-membrane, corresponding to the depressed portions of lung, are converted into fibrous or fibro-cartilaginous tissue; which is sometimes incrusted with calcareous matter, and undergoes constant decrease of size from interstitial contraction. At a still more advanced period, this fibrous disc is absorbed, and nothing remains but a few small fibrous bands, connecting together the well-known irregular mammillary prominences, which the affected parts of the lung present under these circumstances. Meanwhile, the prolongations placed in the sulci, and sunk, as it were, in the interior of the organ, similarly undergo the fibrous, fibro-cartilaginous, or even petrous transformations. Sometimes they retain the character of white fibrinous lamellæ, traversing the adjoining indurated pulmonary tissue in various directions; and occasionally, *all trace of connexion with the external membrane is lost by the perfect closure and adhesion of the outer edges of the sulcus in which they are contained.* The adjoining portion of lung becomes indurated and thickly set with black colouring matter. Now these prolongations of pleural false membrane are, according to M. Fournet, what Laennec took for fibrous cicatrices of tuberculous cavities. Again, these fibrous prolongations sometimes become the seat of a secretion, an excavation forms in their interior, and puriform fluid appears therein; such is the anatomical state which Laennec has, in some instances, taken for the evidence of a cavity in *progress of cicatrization.* Without following M. Fournet seriatim through the very close and plausible train of reasoning, by which these positions are sought to be established, we have given a sufficiently complete outline of his mode of argument to show that his objections to Laennec's theory are not wholly visionary; the reader will, on perusal of the original, find stronger evidence of this fact. The critical scrutiny of the cases published by Laennec and Andral, as affording proofs of the fact of cicatrization, which is appended by our author to the account of his own observations, unquestionably proves that both these writers have ascribed to this process effects otherwise easily explicable, and predicated the tuberculous nature of cavities upon insufficient grounds. Whether M. Fournet's arguments are conclusive of the opinion he desires to substitute for those of his predecessors, further experience must decide. Meanwhile, his general conclusions may be abridged as follows: Pulmonary phthisis is, in extremely rare cases, susceptible of cure during the third stage, but has not been proved to depend upon complete cicatrization of cavities; nor is the mode, in which the cure is effected, yet understood, though it seems more likely to be by conversion of the caverns into fistulæ than by their closure. The tendency of caverns to contraction and obliteration, although a probable circumstance, has not yet received anatomical demonstration; and, finally, the only cases apparently capable of cure are those in which the tuberculous matter occupies a very limited extent of the lung, and gives rise to the formation of a single, or at most of two closely contiguous cavities.

The author shows, in a closing chapter, to what extent legitimate inference from anatomical phenomena supports the notion of the curability of the disease in its crude stage. He commences by examining the truth of Laennec's dogma, that the inevitable tendency of a tubercle is to increase in size and soften,—that the efforts of nature are always pointed in a direction opposed to its cure. This he proves, from Laennec's own lips, to be a perversion of the truth; that author himself actually describes tubercles, bearing all the appearances of having been partially absorbed and replaced by inorganic salts. But to dwell upon what is now a recognized fact—the absorption of tuberculous matter in the lungs—is unnecessary. There is this difference, however, in the opinions of M. Fournet and Dr. Carswell, who has most elaborately traced the anatomical evidences of curability, that the former regards the accidental fibrous tissue, formed occasionally in the substance of the lung, and investing particles or masses of cretaceous matter, as evidences, not of the preexistence of cavities, but simply of that of crude tubercles transformed into that chalky substance. This is, it must be admitted, a very serious modification of previous doctrines, as it transfers the anatomical proofs of cure from the third to the first stage. Our space is exhausted, and we unfortunately cannot condense sufficiently, without weakening their force, the arguments adduced in support of this new view. In respect of the mode of formation of the accidental tissue round tuberculous matter, its successive transformations, its contractile power, and its occasional total disappearance, whereby the pulmonary tissue is left in what, to a superficial observer, appears a state of perfect health, this author follows exactly in the track of Dr. Carswell. Having thus, as he conceives, shown that all the anatomical probabilities are in favour of cure during the first stage, instead of, as has been believed, during the third, M. Fournet emphatically dwells on the encouragement given thereby to our therapeutic efforts; and, in imitation of Sir James Clark, enforces the necessity of general treatment, in the hope of securing such condition of the system at large, as may promote the absorption of the foreign bodies themselves, and obviate a renewed deposition of them.

Before we lay by our pen, we deem it advisable to take a rapid general survey of the work we have just analyzed, of its character, its pretensions, and its originality. If we ask, in the first place, to what extent the tone of the author, and the evidences he adduces in its favour testify to his accuracy of observation, without which quality, in scientific research, all others are as dross, the answer must be of a mixed kind. The cases scattered through and appended to the volume have been minutely observed, and ordinarily bear upon them the stamp of faithful narration; but, on the other hand, how shall we repose implicit trust in the statements of a person, whose passion for distinction is such as to lead him into the error, alike silly and despicable, of appropriating to himself, as an original discoverer, truths unfolded by the researches of others? Again, although he lauds the numerical method with emphasis, he is vastly chary of employing it; and going further even in dogmatism, than authors who use the vague terms "often," "seldom," "little," "much," &c., as expressive of the amount of their experience of particular facts, simply affirms, without hint of their comparative rarity or frequency, that such and such things *are*. When he does state his experience numerically, the smallness of the number of cases referred to is most striking, and justifies the belief, that when he has not dared to use it, their paucity must be singularly subversive of the importance he would fain have attached to his general announcements. The number of subjects examined after death is not even stated in a straightforward manner; and is, rather accidentally than otherwise, discovered to have been only twenty-nine.

The volume is spun out to a needless length by hyperdivision of topics, occasionally by useless wordiness and by frequent repetitions. The preface and some scattered sentences, in which the author's estimate of his own doings appears, prove him affected with the quintessence of the national failing—vanity. These singular outbreaks are, however, rather amusing than otherwise.

But, on the other hand, M. Fournet has given a new character of precision to many phenomena already imperfectly known, added some new ones, and taken a more complete and philosophical view of the system of respiratory signs than any previous author. He has pointed out the route, in his diligent investigation of the two murmurs, to, we doubt not, numerous important additions to semeiology, and taught us how much remains unexplored in this, as was hitherto supposed, almost exhausted field: multum adhuc restat operis, multumque restabit, neque ulli nato . . . . . . præcludetur occasio aliquid adjiciendi. The exact character of his improvements, in the early diagnosis of phthisis, requires explanation. He has assuredly himself learned the means of detecting the disease earlier than his predecessors; some cases of the affection diagnosticated in the very incipient stage (and correctly, as the event proved,) drew this admission from some of the members of the Academy of Medicine. But how has he done this, and to what extent has he taught others the secret? When we knew the contents of his volume only by the preface, we imagined by a number of new signs. So sanguine were we in this respect, that we fancied our future difficulty in the diagnosis of phthisis would be of a very novel kind, that the very multiplicity of decisive signs would almost generate confusion, and,

> "Like a rich armour worn in heat of day,
> That scalds with safety,"

betray us into one evil by the superabundance of protection against another. But we were quickly undeceived. His superior power arises not from the discovery of any new sign, but from his accurate appreciation of signs and symptoms already more or less familiarly known; his careful estimate of their mode of progress; his judicious combination of local signs, symptoms, and general phenomena; and the perfection to which he has brought his acoustic faculty by laborious training. And from this it follows, that they who turn to M. Fournet's volume, in expectation of finding therein a sign or two, in itself easily ascertained and decisive of the existence of the disease, of here discovering, without expenditure of time or exercise of thought, an easy road to a diagnostic millennium, will be sorely disappointed.* On the contrary, before his pages will serve them at the bedside, they must devote their energies to the attainment of a just notion of the value of each category of symptoms, of the progress of these, and tutor their ear into the nicest power of perception.

Finally, M. Fournet has the merit, and it is his highest perhaps, of attempting to turn the attention of his countrymen seriously to therapeutics. Should he succeed in diverting thereto, from its usual sole

* Some persons seem to fancy that the mere application of the stethoscope to the chest is quite enough for purposes of diagnosis, that this, by a sort of mesmeric power, discloses all that goes on beneath. And we must in candour admit that these individuals work wonders with the instrument. At least, we lately met a gentleman in consultation, who had hardly placed the stethoscope close to the *upper and anterior edge of the precordial region* when he detected *pleurisy*, though the patient, whose pleura was as sound as his own, really laboured under nothing more than *capillary bronchitis of the posterior bases* of both organs. Unfortunately for persons of this stamp, the stethoscope is not a thinking, speaking, diagnosing being, but simply

> "Ist wie das todte Sprachrohr, das den Schall
> Empfängt und wiedergiebt, und selbst nicht höret."

object, pure pathological anatomy, even a tithe of the energy the French enquirers are wont to exhibit, something may be hoped from his innovation. And while it will afford no small satisfaction to the profession in this country to find that the branch of medicine, to which they have always directed their chiefest attention, begins to assert its due rank among their continental brethren, the distinguished physician, from whose work on Phthisis M. Fournet's scheme of treatment is derived, must behold with honest pride the sterling improvement to which the dissemination of his labours shall eventually give rise.

---

